# High water use efficiency due to maintenance of photosynthetic capacity in sorghum under water stress

**DOI:** 10.1093/jxb/erae418

**Published:** 2024-10-08

**Authors:** Yazen Al-Salman, Francisco Javier Cano, Emma Mace, David Jordan, Michael Groszmann, Oula Ghannoum

**Affiliations:** ARC Centre of Excellence for Translational Photosynthesis, Canberra, ACT, Australia; Hawkesbury Institute for the Environment, Western Sydney University, Richmond, NSW, Australia; ARC Centre of Excellence for Translational Photosynthesis, Canberra, ACT, Australia; Instituto de Ciencias Forestales (ICIFOR-INIA), CSIC, Carretera de la Coruña km 7.5, 28040, Madrid, Spain; ARC Centre of Excellence for Translational Photosynthesis, Canberra, ACT, Australia; Queensland Alliance for Agriculture and Food Innovation, Hermitage Research Facility, University of Queensland, Warwick, QLD, Australia; Department of Agriculture and Fisheries, Agri-Science Queensland, Warwick, QLD, Australia; ARC Centre of Excellence for Translational Photosynthesis, Canberra, ACT, Australia; Queensland Alliance for Agriculture and Food Innovation, Hermitage Research Facility, University of Queensland, Warwick, QLD, Australia; Department of Agriculture and Fisheries, Agri-Science Queensland, Warwick, QLD, Australia; ARC Centre of Excellence for Translational Photosynthesis, Canberra, ACT, Australia; Division of Plant Sciences, Research School of Biology, The Australian National University, Acton, ACT 2601, Australia; Grains Research and Development Corporation (GRDC), Barton, ACT 2600, Australia; ARC Centre of Excellence for Translational Photosynthesis, Canberra, ACT, Australia; Hawkesbury Institute for the Environment, Western Sydney University, Richmond, NSW, Australia; University of Essex, UK

**Keywords:** C_4_ crops, genotypic variation, hydraulic conductance, sorghum, stomatal conductance, water stress, water use efficiency

## Abstract

Environmental change requires more crop production per water use to meet the rising global food demands. However, improving crop intrinsic water use efficiency (iWUE) usually comes at the expense of carbon assimilation. Sorghum is a key crop in many vulnerable agricultural systems with higher tolerance to water stress (WS) than most widely planted crops. To investigate physiological controls on iWUE and its inheritance in sorghum, we screened 89 genotypes selected based on inherited haplotypes from an elite line or five exotics lines, containing a mix of geographical origins and dry versus milder climates, which included different aquaporin (AQP) alleles. We found significant variation among key highly heritable gas exchange and hydraulic traits, with some being significantly affected by variation in haplotypes among parental lines. Plants with a higher proportion of the non-stomatal component of iWUE still maintained iWUE under WS by maintaining photosynthetic capacity, independently of reduction in leaf hydraulic conductance. Haplotypes associated with two AQPs (SbPIP1.1 and SbTIP3.2) influenced iWUE and related traits. These findings expand the range of traits that bridge the trade-off between iWUE and productivity in C_4_ crops, and provide possible genetic regions that can be targeted for breeding.

## Teaser

High water use efficiency is linked to maintenance of photosynthesis independently of leaf hydraulic conductance in sorghum lines with different aquaporin-associated haplotypes.

## Introduction

Food security amid water scarcity is one of the key global challenges of the 21st century (UNCTAD, 2011). Sorghum (*Sorghum bicolor*) is globally important for fuel, fibre, food (Borrell *et al.*, 2014b), and animal feed (George-Jaeggli *et al.*, 2017). Sorghum, a C_4_ species, was first domesticated in Africa, where it remains a key staple crop in the arid and semi-arid areas of sub-Saharan Africa, a region experiencing a rapid rise in population (Dillon *et al.*, 2007; Borrell *et al.*, 2014a). Such environments are heavily dependent on rainfall, which are expected to show more erratic patterns with climate change (Rippke *et al.*, 2016). With intensifying water scarcity, more attention is being paid to crop productivity per unit of transpired water (Passioura, 2006). This characteristic is termed transpiration efficiency or water use efficiency (WUE) (Passioura, 1977). At the leaf level, the physiological control of WUE is quantified as the ratio of leaf carbon assimilation (*A*_n_) to stomatal conductance to water vapour (*g*_s_), and termed intrinsic water use efficiency (iWUE).

Selecting for higher iWUE in breeding programmes of C_4_ crops has been challenging for a number of reasons. First, iWUE is a complex trait with multiple physiological components contributing to the variations in *A*_n_ and *g*_s_ (Condon *et al.*, 2004). Secondly, there is a potential lack of heritable WUE-related traits that can be easily screened (Hammer *et al.*, 1997). Proxies for iWUE in C_3_ crops such as carbon isotope discrimination are not easily applicable in C_4_ counterparts (Condon and Richards, 1992; Henderson *et al.*, 1998; Rebetzke *et al.*, 2002; von Caemmerer *et al.*, 2014; Ellsworth and Cousins, 2016; Ellsworth *et al.*, 2020). Hence, finding genetic variation in iWUE among C_4_ crops has mainly depended on gas exchange parameters (Xin *et al.*, 2009). Consequently, improving iWUE in C_4_ crops requires a better understanding of the mechanisms leading to genetic variation in gas exchange and iWUE (Jackson *et al.*, 2016).

Achieving higher iWUE can come at the expense of photosynthesis and biomass production (Martin *et al.*, 1999; Condon *et al.*, 2004; Passioura, 2006; Blum, 2009). This is because increases in iWUE may result from restricting water use via stomatal closure, which usually occurs during water stress (WS). However, if the leaf can still maintain a high photosynthesis rate at lower intercellular [CO_2_] (*C*_i_) due to stomatal closure, then iWUE increases can benefit biomass production when water is scarce and allow water to remain in the soil for later phenological stages (Sinclair *et al.*, 2005; Vadez, 2019; Srivastava *et al.*, 2024). Still, higher *g*_s_ and water use associated with high photosynthesis has led to higher yields in a number of crops under both WS (Blum *et al.*, 1982; Sanguineti *et al.*, 1999; Araus *et al.*, 2003; Vijayaraghavareddy *et al.*, 2020; Ouyang *et al.*, 2022; de Oliveira *et al.*, 2023) and well-watered (WW) (Reynolds *et al.*, 1994; Fischer *et al.*, 1998; Horie *et al.*, 2006) conditions. Therefore, a key challenge is to understand how to screen for greater iWUE without sacrificing greater productivity, especially under WS (Leakey *et al.*, 2019; de Oliveira *et al.*, 2022, 2023).

iWUE depends on the *A*_n_–*g*_s_ relationship, which is almost linear at low to moderate *g*_s_, and reaches a plateau at high *g*_s_ (Wong *et al.*, 1979; Gilbert *et al.*, 2011). Consequently, *A*_n_ and *g*_s_ contribute different proportions to iWUE depending on their operational position along the *A*_n_–*g*_s_ curve (Ghannoum, 2016). When comparing different plants, high iWUE may be due to higher *A*_n_, and/or lower *g*_s_ (Leakey *et al.*, 2019). The operation of the CO_2_-concentrating mechanism (CCM) in C_4_ leaves leads to the saturation of *A*_n_ at lower *C*_i_ than in C_3_ plants, and hence low *g*_s_, which means that operating with high *g*_s_ may lose water without improving *A*_n_ (Srivastava *et al.*, 2024). On the other hand, some crop varieties can sustain high iWUE due to higher photosynthetic capacity per given *C*_i_, and Gilbert *et al.* (2011) proposed a method to screen for variation in iWUE associated with stomatal or non-stomatal components of *A*_n_ applied to soybean (C_3_ dicot) and later applied by Li *et al.* (2017) in sugarcane (C_4_ monocot). Finding such varieties is agronomically beneficial as it would alleviate the often-negative relationship between iWUE and photosynthesis or productivity.

The contribution of plant or leaf water status to WS responses can also be an important determinant of the trade-off between iWUE and photosynthesis. During WS, the hydraulic flux of water from the soil to the sites of transpiration within the leaves is often reduced, leading to a decrease in plant (*K*_plant_) and leaf (*K*_leaf_) hydraulic conductances. Consequently, leaves close stomata to maintain cell turgor and metabolism, and to reduce the risk of catastrophic hydraulic failure (Meinzer and Grantz, 1990; Mott and Franks, 2001; Meinzer, 2002; Brodribb *et al.*, 2003), which also reduces CO_2_ supply for photosynthesis. One by-product of selecting for high iWUE under WS is obtaining varieties that favour water conservation in the soil, sometimes at the cost of photosynthesis (Choudhary *et al.*, 2013; Choudhary and Sinclair, 2014). This strategy often selects varieties with low *K*_leaf_ or that reduce *K*_leaf_ significantly during WS and especially at high vapour pressure deficit (VPD). However, lower *K*_leaf_ negatively impacts photosynthesis either directly, or indirectly via reducing *g*_s_ and hence *C*_i_. Hence, screening for variation in hydraulic responses to WS can identify varieties that maintain *A*_n_ despite reduced *C*_i_ under WS, attaining higher iWUE.

A possible target that link photosynthesis, water relations, and iWUE are aquaporins (AQPs) (Vadez *et al.*, 2014; Reddy *et al.*, 2015). AQPs are channel proteins embedded in the lipid bilayer of plant cellular membranes. AQPs strongly influence the flow of water and ions within the leaf, affecting physiological parameters such as *K*_leaf_ and iWUE (Maurel *et al.*, 2015), including in sorghum (Choudhary *et al.*, 2013; Hasan *et al.*, 2015; Liu *et al.*, 2015; Hasan *et al.*, 2017; Zhang *et al.*, 2019). More importantly, several AQPs in plants have been shown to be key CO_2_ transporters (sometimes called cooporins) especially across the plasma membrane (Groszmann *et al.*, 2017). Hence, they could hypothetically increase CO_2_ or H_2_O supply to the sites of carboxylation without increasing *g*_s_.

Screening for variation in physiological traits is laborious and time-consuming, and requires an extensive number of genotypes. We explored the rich genetic resources that are available for sorghum (Mace *et al.*, 2019), using variations in genomic regions associated with different AQP alleles (haplotypes) from a sorghum nested association mapping (NAM) population (see the Materials and methods). We curated >80 genotypes and grew them under two watering regimes to assess the degree of variation of iWUE and other plant traits in closely similar sorghum genotypes under WS and to use that variation to test the following hypotheses: (i) partitioning the stomatal and non-stomatal components of iWUE within this diversity will reveal genotypes that achieve high iWUE under WS by maintaining photosynthesis; (ii) achieving high iWUE under WS due to maintenance of photosynthesis will be underpinned by maintenance of *K*_leaf_ and leaf water status; and, finally, (iiii) the maintenance of hydraulics and photosynthesis under WS will be linked to certain AQPs and their related haplotypes.

## Materials and methods

### Genotype selection

**Fig. 1.**
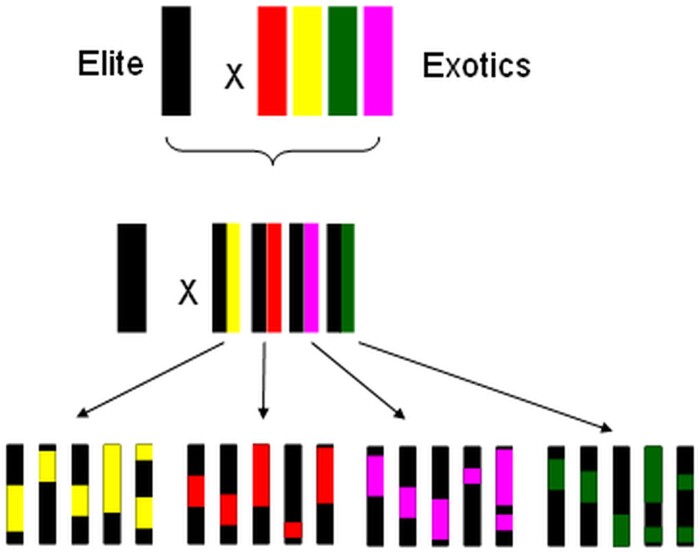
A simplified illustration of how recombinant inbred lines (RILs) are produced using nested association mapping (NAM).

The genotypes used in this study are a part of a NAM population (Jordan *et al.*, 2011; Tao *et al.*, 2020). NAM is a type of selective breeding that allows for statistical robustness while retaining diversity of parental lines. NAM maintains some allelic diversity by breeding (and backcrossing) recombinant inbred lines (RILs) from multiple parents with a single parent as a reference line (Fig. 1). Hence, the progeny share most of their genetic material, and phenotypic differences can be quickly linked to specific genetic regions. Genotypes used in our study came from a sorghum NAM population that comprises an elite parental line R937945-2-2 (recurrent parent, RP) crossed with >100 exotic lines with geographical or racial diversity (non-recurrent parent, NRP). The F_1_ progeny were backcrossed with the elite parent to produce BC_1_F_1_ populations. BC_1_F_1_ genotypes compromise ~22–25% exotic (NRP) line genome, with the rest being RP background (Fig. 1). Individual BC_1_F_1_ populations are genotyped using high-density single nucleotide polymorphism (SNP) markers providing profiles of the exact exotic chromosomal segments, giving us information on what genes are coded for in the 22–25% NRP portion of the genome, and what genes are coded for in the remaining RP section of the genome. In addition to this resource, whole-genome sequencing is available for many of the exotic parental lines and the elite line (Mace *et al.*, 2013).

We screened this sorghum NAM population for genes of eight AQPs to select lines carrying non-synonymous SNP alleles of those genes. Specifically, the subpopulation was screened to identify individual lines with chromosomal segments harbouring the elite (RP) AQP allele (RP-haplotype) or the exotic (NRP) AQP allele (NRP-haplotype) of a specific AQP. The final 89 lines chosen were derived from five exotics (NRPs) containing a mix of geographical origins, with specific focus on a mix of dry versus milder climates with the idea that these would have greater extremes in the traits of interest due to necessary adaptations to their climate of origin (Table 1). This approach allowed us to create subpopulations within the 89 genotypes through focusing on one of the eight AQPs, with each subpopulation containing two sets of genotypes, a set (>5) of genotypes containing the RP-haplotype for that AQP, and a set containing the NRP-haploype. Hence, any phenotypic difference when comparing RP or NRP haplotypes associated with a certain AQP may be due to the specific AQP allele that characterizes the RP or NRP haplotype or from the accompanying genes from that chromosomal segment (haplotype), creating a link between phenotype and genotype.

**Table 1.** The elite (RP) and exotic (NRP) parents used in the NAM breeding programme

ID	Origin	Description
**SC103-14E**	South Africa	Originates from hot, dry regions of Ethiopia and Sudan.
**Ai4**	China breeding programme	Breeding variety not known for drought resistance.
**FF_RT×7000**	US breeding programme	High yielding line that uses a lot of water and grows very well; drought sensitive.
**QL12**	Australia breeding programme	Australian breeding variety known for drought tolerance
**IS9710**	Sudan	Known for high transpiration efficiency; originated in dry regions.
**R931945-2-2**	Australia breeding programme	Elite parental line, the RP.

Information about the parents and the production of the NAM population can be found in Jordan *et al.* (2011).

### Plant culture

Cylindrical pots (8 litre) were used to allow ample space for root development before implementation of the water stress treatment. The pots were adjusted to similar weight (1.5 kg) by adding gravel (100–300 mm diameter), then the same amount of soil was added to all pots. Fly screen mesh (aluminium insect screen) was added to the bottom of the pots to minimize soil seeping through pot drainage holes. The potting mix was made of soil, sand, and decomposed bark. It has large particle size for good drainage and root development. Granulated fertilizer (Osmocote Plus Organics All Purpose Fertiliser, Scotts Miracle-Gro Company, Marysville, OH, USA) was pre-mixed with the soil, with more fertilizer added in the lower half of the pot where more roots will develop as the plant grows. To each pot, 3.5 kg of soil was added, making the total pot weight 5 kg. We left 2–3 cm at the top of the pot empty, making the volume of the soil filled 7.5 litres.

Seeds were directly sown into the upper soil layer in October 2019. Plants germinated and grew in a naturally lit, controlled-environment greenhouse (Plexiglas Alltop SDP 16; Evonik Performance Materials, Darmstadt, Germany) at the Hawkesbury Institute for the Environment, Western Sydney University, Richmond, New South Wales, Australia (–33.612032, 150.749098). The ambient temperature was set at 30 °C during the day period, with night temperatures set at 18 °C. There was a 2 h period at 24 °C between the temperature transitions. The day temperature started at 08.00 h and night temperature at 20.00 h, when sunrise was about 05.00–06.00 h and sunset at 19.00–20.00 h, reaching midday maximums of 34–35 °C and midday relative humidity of 40–50% (Supplementary Fig. S1). CO_2_ concentration was kept at ambient levels. Due to the large number of plants, we needed three identical and adjacent greenhouse chambers (8 m long×3 m wide×5 m tall), which contained both WW and WS pots, and pots were swapped between the three chambers every 2 weeks during growth in a randomized fashion. Chamber conditions were monitored via a data logger (Tinytag plus 2, Omni Instruments) hung in the middle of the room at 2 m height. Light levels were monitored occasionally using a light meter and were 1500 µmol m^–2^ s^–1^ at midday on sunny days at plant height level at measurement time (~2 m from the ground).

### Watering treatments

All plants were well watered for the first 6 weeks of growth when half of the plants by genotype were subjected to WS and the other half continued under WW conditions. When plants were 5 weeks old, pots were weighed in the late evening (*W*_evening_), then watered at dusk and weighed again in the morning (*W*_morning_). This allowed pots to drain excess water with minimal loss via evaporation during the night and determine pot weight at field capacity (FC) by the repetition of this routine over three consecutive sunny days and taking the average of *W*_morning_. On each morning, we also measured the volumetric soil water content (VSWC) with a sensor (Campbell Scientific, Logan, UT, USA) on each pot after measuring *W*_morning_. FC was 13–15% for our soil. The difference between pot weight at FC and pot weight before watering in the evening (*W*_morning_–*W*_evening_) represented the amount of water transpired by each plant during the day under WW conditions. After 6 weeks of growth, watering was withheld from half of the pots (WS; water stress treatment), while the other half continued to be watered at FC (WW; well-watered treatment). Stomatal conductance was monitored in WS plants until it reached ~0.1 mol m^–2^ s^–1^ or less at saturating light, with the plant also showing signs of wilting. When conductance reached the required level, and signs of wilting appeared, the VSWC was ~5% for most pots. At this point, we measured pot weight as described before to establish the amount of water lost to transpiration by the plants in the WS treatment (~50 ml). Three-fold this amount of water, equivalent to total plant transpiration during the day in the WS treatment for 3 d, was added every 3 d to the WS pots. Hence, plants under WS got just enough water for replacement of water loss via daytime evapotranspiration, and we ensured that water status of WS plants was not influenced by recent watering by the delaying of measurements to the third day after watering.

The two watering regimes were maintained until the end of the experiment, constituting the two treatments: WW—FC; WS—50 ml every day or 150 ml every 3 d. The impact of WS was visible 2 weeks after water withholding for most genotypes (plants were 8 weeks old). There were three replicates (pots) per genotype and water treatment. Hence, each genotype had six pots in total, with three for each treatment (*n*=3), except for the elite parent R937945-2-2 (the RP) which had six pots per treatment (*n*=6).

### Time of measurements and sampling

Plants were sampled between weeks 9 and 12 after germination, when they had 10–12 fully expanded leaves. WS plants were measured at least 3 weeks after the onset of the drought treatment. In total, sampling lasted for about a month (mid-December 2019 to mid-January 2020), which represents the peak of the Australian summer. Priority for physiological sampling was given to plants at the booting stage so that all plants were measured before or at the start of flowering.

### Midday leaf gas exchange

Midday leaf gas exchange rates were measured between 10.00 h and 14.00 h on sunny days. The photoperiod was 14–15 h and solar midday was at around 13.00–13.30 h. A Li-6400XT infrared gas analyser with an LED light source and an area of 6 cm^2^ (LiCor Biosciences, Lincoln, NE, USA) was used to obtain light-saturating rates of CO_2_ assimilation (*A*_n_), stomatal conductance to water vapour (*g*_s_), and transpiration flux (*E*); cuvette conditions were set at: 30 °C block temperature, flow rate of 500 µmol m^–2^ s^–1^, photosynthetic photon flux density (PPFD) of 2000 µmol m^–2^ s^–1^ (10% blue light), ambient CO_2_ concentration set to 400 ppm using a CO_2_ cylinder mixer, and relative humidity of 40–60%. The leaf was inserted into the gas exchange cuvette under those conditions, avoiding the midrib and with the entire 6 cm^2^ area of the cuvette filled. The leaf was left to acclimate to those conditions until gas exchange and CO_2_ concentration in the substomatal cavity (intercellular CO_2_, *C*_i_) stabilized. iWUE was calculated as the ratio of *A*_n_ to *g*_s_. All measurements were taken from the middle of the youngest fully expanded leaf (YFEL) of the plant, corresponding to the 9th–12th leaf depending on genotype. The ambient light level at the YFEL was ~500 µmol m^–2^ s^–1^.

### Leaf water potential and hydraulic conductance

A leaf adjacent to the gas exchange leaf was used to measure midday leaf water potential (Ψ_midday_) using a Scholander-type pressure chamber (Model 1505D Pressure Chambers, PMS Instrument Company, Albany, OR, USA). The leaf below the Ψ_midday_ leaf was covered with cling wrap and aluminium foil to prevent transpiration and allow the leaf to equilibrate for at least 6 h (usually they were covered before gas exchange measurements started or the day before and collected at the end of the day and taken to the lab). This leaf was then used to estimate midday stem water potential (Ψ_stem_). Pre-dawn leaf water potential (Ψ_pre-dawn_) was sampled on different leaves before daybreak, usually taking leaves in the lower canopy. In each case, the leaf was cut at the ligule and placed in a plastic bag that was exhaled into before sealing. The bags were stored in ice boxes, then transported from the greenhouse to the lab where leaf water potentials were measured within 1–2 h of excision.

Leaf hydraulic conductance was calculated as shown in Simonin *et al.* (2015):


$$\begin{array}{l} K_{leaf}=\frac{E}{(\Psi_{stem}-\Psi_{midday)}} \end{array}$$ (1)

where *E* refers to the leaf transpiration rate at the time of excision, estimated by measuring incident PPFD at the time of leaf excision and then *E* at that PPFD level estimated from light–response curves conducted on the same plant. Soil-to-leaf hydraulic conductance (referred to as plant hydraulic conductance, *K*_plant_) was calculated as shown in Robson *et al.* (2012):


$$\begin{array}{l} K_{plant}=\frac{E}{({\;\Psi }_{pre\text{-}dawn}-\Psi_{midday)}} \end{array}$$ (2)

Leaf hydraulic resistance (*R*_leaf_) was calculated as 1/*K*_leaf_. Hydraulic resistance of the rest of the plant (*R*_rest_) was calculated as (1/*K*_plant_)–*R*_leaf_.

### Plant and leaf morphology

Leaf width (LW) was measured at the same leaf area where gas exchange measurements were made. Leaf length (LL) was also measured. Leaf thickness (LT) was measured using a Photosynq Multispec (Photosynq, East Lansing, MI, USA). At the end of the experiment and before biomass harvest, plant height (PH) and number of leaves (LN) of each plant were recorded. In this same leaf and area of leaf for which we measured gas exchange, we collected three leaf discs of 0.5 cm^2^ each to measured leaf mass per area (LMA) and relative water content (RWC). First, we placed leaf discs inside Eppendorf tubes in ice to quickly measure FW in a four positions balance, then we added distilled water and kept them in darkness and at 4 °C overnight before measuring the turgid weight (TW) again. Finally leaf discs were placed inside an oven at 65 °C for 48 h to measure DW. LMA was calculated as DW leaf discs area (g m^–2^) and RWC as: (FW–DW)/(TW–DW). Plants were harvested after 95–100 d, and total above-ground biomass was separated into panicle and vegetative (i.e. leaves and stem) to dry in an oven at 40 °C for 10 d before measuring dry biomass, but we present above-ground biomass in the data below as encompassing panicles and vegetative.

### Relative chlorophyll content and quantum efficiency of PSII

Relative chlorophyll content was estimated by a SPAD meter that is embedded in the Photosynq Multispeq (Kuhlgert *et al.*, 2016). SPAD meters measure absorbance at 650 nm and 940 nm, and then relative values for chlorophyll content are produced. The Multispec was also used to record the quantum efficiency of PSII (ΦPSII) using a pulse-amplitude fluorometer at ambient light. Measurements were conducted on the same leaf as used for gas exchange.

### Components of intrinsic water use efficiency

To partition the relative contribution of *A*_n_ and *g*_s_ to variation in iWUE in our population, the approach of Gilbert *et al.* (2011) was used as modified by Li *et al.* (2017). Briefly, because of the curvilinear relationship between *A*_n_ and *g*_s_, it is expected that *A*_n_ and *g*_s_ will contribute in different proportions to iWUE depending on the position of the genotype along the curve and with respect to the mean population value.

From each measurement of gas exchange, we constructed a curve of iWUE versus *g*_s_ encompassing all treatments. We then calculated the average iWUE of all measurements for each treatment. To obtain variation in iWUE due to *g*_s_ (ΔiWUE_*g*s_), the iWUE expected if iWUE was calculated from our reference curve (iWUE versus *g*_s_, i.e. constant *A*_n_) and then ΔiWUE_*g*s_ was expressed as the deviation of the calculated iWUE from the population mean of iWUE for that treatment. This results in a value that highlights how impactful *g*_s_ was in deviating that genotypic iWUE from the population mean assuming fixed *A*_n_ (a negative value for ΔiWUE_*g*s_ would mean that a *g*_s_ increase for that genotype reduced iWUE by that level compared with the mean). Variation in iWUE due to *A*_n_ (ΔiWUE_pc_; where pc stands for photosynthetic capacity)—the non-stomatal component—was then calculated as the difference between the actual measured iWUE and calculated iWUE based on *g*_s_ variation. Basically ΔiWUE_pc_ represents the remaining ‘difference’ between the population mean iWUE and genotypic iWUE that was not covered by ΔiWUE_*g*s_ This means that variation in these two components can highlight how each of *g*_s_ and *A*_n_ contribute to iWUE. For example, for a given genotype, if ΔiWUE_*g*s_ is small but ΔiWUE_pc_ is large (both positive), it means that iWUE is higher than the population mean because of higher photosynthesis mainly and lower conductance secondarily (see Supplementary Fig. S2 for an illustration). We also compared ΔiWUE_*g*s_ and ΔiWUE_pc_ values if taken from a reference curve that is based on a reference genotypes, and it showed complete agreement (*R*=0.98, Supplementary Fig. S3).

### Calculating the magnitude of change in *A*_n_ and *C*_i_ in response to water stress

To investigate how genotype response to WS enables the achievement of high iWUE by amplifying one of its two components highlighted earlier, we calculate the ‘degree of change’ in a hypothetical *A*_n_–*C*_i_ curve based on genotype mean value change between WW and WS (Rowland *et al.*, 2023). This method estimates both the magnitude of the change and the direction of the phenotypic change vector (the angle) between two contrasting environments. The change in the angle, θ, represents change in trait covariation, in our case the dependence of *A*_n_ on *C*_i_. Small changes in θ would indicate a large decrease in *C*_i_ but a small decrease in *A*_n_, pointing to drought resilience, meaning the achievement of higher iWUE due to stomatal closure but also maintenance of photosynthesis rates. A large θ would indicate a combined plummeting in *A*_n_ with *C*_i_, meaning that iWUE would increase less due to photosynthesis and more due to stomatal closure under WS (see the Results for further clarification).

### Genetic variation

Broad-sense heritability was calculated as in Li *et al.* (2017):


$$\begin{array}{l} H_{b}^{2}=\frac{\Sigma_{g}^{2}}{\Sigma_{p}^{2}} \end{array}$$ (3)

where σ_g_^2^ and σ_p_^2^ are the genotypic and phenotypic variances, respectively. σ_g_^2^ was obtained as the square of the mean from the ANOVA output. σ_p_^2^ was calculated as:


$$\begin{array}{l} \Sigma_{p}^{2}=\Sigma_{g}^{2}+\frac{\Sigma_{g\times treatment}^{2}}{number\;of\;treatments}+\frac{\Sigma_{e}^{2}}{number\;of\;replicates} \end{array}$$ (4)

where σ_g×treatment_^2^ and σ_e_^2^ are the genotype×treatment interaction and error variances, respectively. σ_g×treatment_^2^ was obtained as the mean squared of the genotype×treatment interaction and σ_e_^2^ was obtained as the square of the mean residual error. Because the heritability analysis encompasses both treatments,he number of replicates was standardized as 5 (as opposed to 6; 3 WW and 3 WS) to account for genotypes not in both treatments. The genotypic coefficient of variation (GCV) and the phenotypic coefficient of variation (PCV) were calculated as:


$$\begin{array}{l} GCV=\frac{\Sigma_{g}}{mean}\times 100 \end{array}$$ (5)

$$\begin{array}{l} PCV=\frac{\Sigma_{p}}{mean}\times 100 \end{array}$$ (6)

where σ_g_ and σ_p_ are the genotypic and phenotypic standard deviation. The mean refers to the mean of all the measurements across treatments for the variable in question. For the mean value of iWUE_*g*s_ and iWUE_pc_ where averages are near zero or negative (because these values are expressed as deviations from the average of all observations), the value used for mean was that for iWUE.

### Statistical analyses

**Fig. 2.**
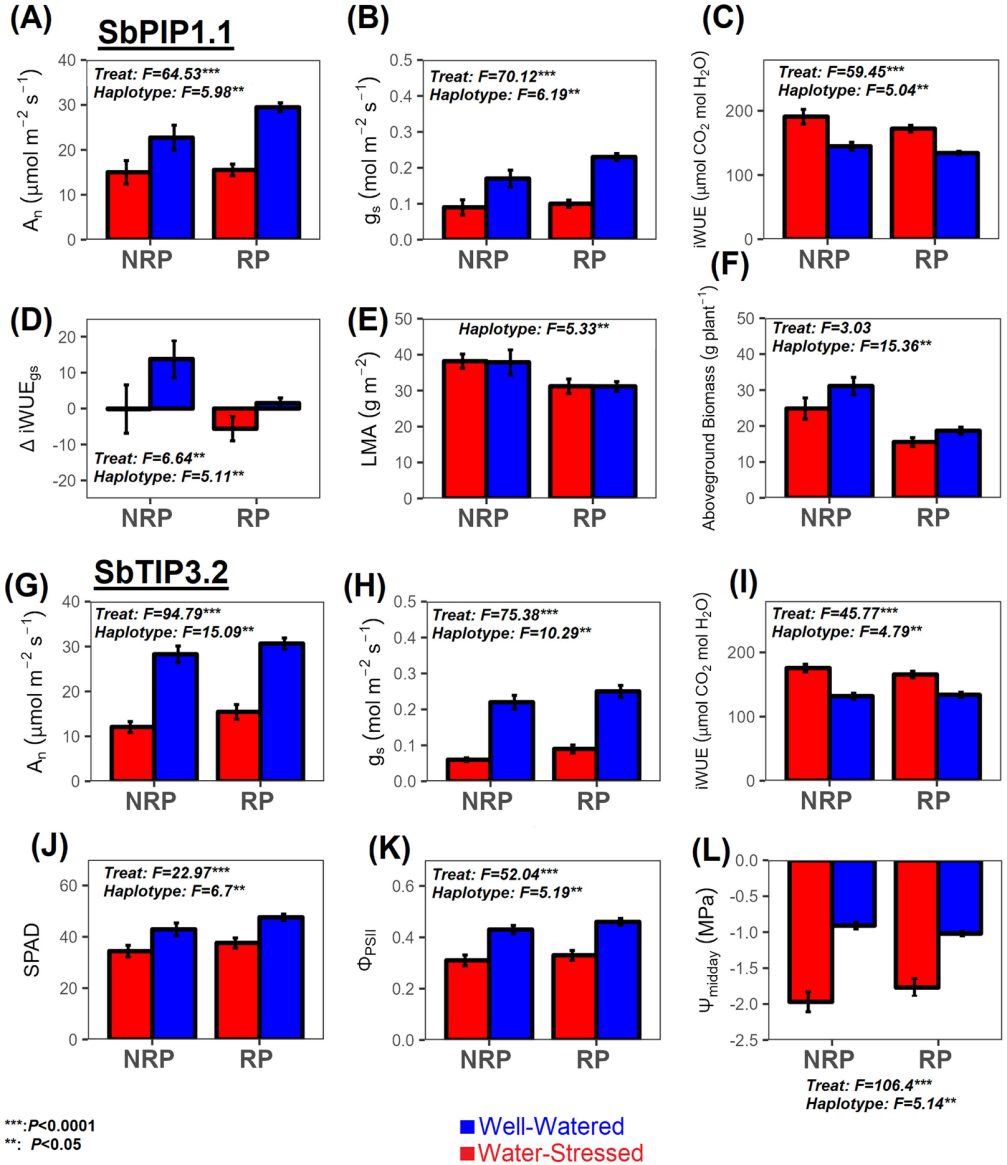
Bar charts showing the effects of two aquaporin haplotypes on key traits. The two AQPs shown are those that showed significant differences in several key traits between haplotype genotype populations, with the full analysis for all AQPs shown in Table 2. Each bar represents the mean of all individual replicates belonging to the genotypes of that population (*n*=18–63; see Supplementay Table S3 for the number of genotypes for each haplotype×treatment combination). Statistics shown are the result of ANOVA test and post-hoc Tukey test. Bars that share the same letter have no significant differences between them at *P*<0.05. For information about the approach to genotype selection, see the Materials and methods. Each population (RP and NRP) refers to a set of genotypes that have inherited the AQP haplotype block either from the elite parent (RP) or from the exotic parent (NRP). The traits shown are (A and G) carbon assimilation rate (*A*_n_); (B and H) stomatal conductance (*g*_s_); (C and I) intrinsic water use efficiency (iWUE); (D) variation in iWUE due to *g*_s_ (ΔiWUE_*g*s_); (E) leaf mass per area (LMA); (F) above-ground biomass; (J) leaf chlorophyll content (SPAD); (K) operating efficiency of PSII (Φ_PSII_); (L) midday leaf water potential (Ψ_midday_).

Statistical analysis and data visualization were performed using R software (R Core Team, 2020). Normality was checked by plotting a generalized linear model and inspecting residual plots. ANOVA and multiple ANOVA (MANOVA) were carried out using linear mixed-effects models (package nlme), with replicate and genotype as the random variable, respectively, and the fixed variables being AQP haplotype×water treatment to get the *P*-value associated with the model (Fig. 2 and Table 2, respectively). Variance within groups was performed afterwards using a post-hoc Tukey test. Regression analysis was carried in R using linear modelling (lm). A Pearson product moment correlation analysis was performed to test statistical significance of relationships at *P*<0.05 and obtain correlation coefficients *R* (which were then converted into *R*^2^).

**Table 2.** Summary of *P-*values from the mixed effect MANOVA of the parameters

Exotic parent	Aquaporin	Comparison	df	*A*_n_	*g*_s_	iWUE	ΦPSII	Ψ_midday_	*K*_leaf_	LMA	*R*_leaf_	*R*_rest_	SPAD	Tot Biom	ΔiWUE_*g*s_	ΔiWUE_pc_
FF_RT×7000	PIP 2.7	Population	1	ns	ns	ns	ns	ns	ns	ns	ns	ns	ns	ns	ns	ns
		Treatment	1	**0.0001**	**0.0001**	**0.0001**	ns	**0.0001**	**0.08**	ns	**0.0075**	**0.0015**	**0.06**	ns	**0.04**	**0.03**
		Population×Treatment	1	ns	ns	ns	ns	ns	ns	ns	ns	**0.053**	ns	ns	ns	ns
QL12	TIP 1.1	Population	1	ns	ns	ns	ns	ns	ns	ns	ns	ns	ns	ns	ns	ns
		Treatment	1	**0.0001**	**0.0001**	**0.0001**	**0.0001**	**0.0001**	**0.0017**	ns	**0.0002**	**0.0001**	**0.0001**	ns	ns	ns
		Population×Treatment	1	ns	ns	ns	ns	ns	ns	ns	ns	ns	ns	ns	ns	ns
QL12	TIP 3.2	Population	1	**0.0011**	**0.0049**	**0.04**	**0.035**	**0.036**	ns	ns	ns	**0.018**	**0.019**	ns	ns	ns
		Treatment	1	**0.0001**	**0.0001**	**0.0001**	**0.0001**	**0.0001**	**0.0001**	ns	**0.0003**	**0.0001**	**0.0001**	ns	ns	ns
		Population×Treatment	1	ns	ns	ns	ns	**0.07**	**0.03**	ns	**0.051**	ns	ns	ns	ns	ns
SC103-14E	TIP 4.3 and 4.4	Population	1	ns	ns	ns	**0.058**	ns	ns	ns	ns	ns	**0.044**	ns	ns	ns
		Treatment	1	**0.0001**	**0.0001**	**0.0001**	**0.0001**	**0.0001**	ns	ns	**0.019**	**0.012**	**0.05**	**0.0036**	ns	ns
		Population×Treatment	1	ns	ns	ns	ns	ns	ns	ns	ns	ns	ns	ns	ns	ns
IS9710	TIP 2.1	Population	1	ns	ns	ns	ns	ns	ns	ns	ns	ns	ns	ns	ns	ns
		Treatment	1	**0.0001**	**0.0001**	**0.0001**	**0.0001**	**0.0001**	**0.0001**	ns	**0.0066**	**0.011**	**0.0001**	**0.06**	**0.002**	**0.001**
		Population×Treatment	1	ns	ns	ns	ns	ns	ns	ns	ns	ns	ns	ns	ns	ns
Ai4	PIP 2.10	Population	1	ns	ns	ns	ns	ns	ns	ns	ns	ns	ns	ns	ns	ns
		Treatment	1	**0.0001**	**0.0001**	**0.0001**	**0.0001**	**0.0001**	**0.0001**	ns	**0.039**	ns	**0.0001**	**0.02**	ns	ns
		Population×Treatment	1	0.05	ns	ns	ns	ns	ns	ns	ns	ns	ns	ns	ns	ns
IS9710	PIP 1.6	Population	1	ns	ns	ns	ns	ns	ns	ns	ns	ns	ns	ns	ns	ns
		Treatment	1	**0.0001**	**0.0001**	**0.0001**	**0.0001**	**0.0001**	**0.0048**	ns	**0.0071**	**0.0081**	**0.0001**	**0.03**	**0.02**	**0.015**
		Population×Treatment	1	ns	ns	ns	ns	ns	ns	ns	ns	ns	ns	ns	ns	ns
IS9710	PIP 1.1	Population	1	**0.02**	**0.02**	**0.03**	ns	ns	ns	**0.03**	ns	ns	ns	**0.0007**	**0.035**	ns
		Treatment	1	**0.0001**	**0.0001**	**0.0001**	**0.0001**	**0.0001**	**0.0044**	ns	**0.0043**	**0.0086**	**0.0001**	**0.03**	**0.012**	**0.01**
		Population×Treatment	1	ns	ns	ns	ns	ns	ns	ns	ns	ns	ns	ns	ns	ns

Bold indicates *P*-values <0.05). Population refers to the comparison between genotypes that have the recurrent parent (RP) haplotype for the AQP and genotypes that have the non-recurrent parent (NRP) haplotype for that AQP. Treatment means the watering level: well-watered and water-limited. Both comparison have two levels (df=1). *n* (6–63), for the number of independent genotypes per haplotype group for each specific AQP see Supplementary Table S3.

Abbrevations: *A*_n_, carbon assimilation rate; *g*_s_, stomatal conductance; iWUE, instantaneous water use efficiency; ΦPSII, operating quantum yield of PSII; Ψ_midday_, midday leaf water potenial; *K*_leaf_, leaf hydraulic conductivity; LMA, leaf mass per area; *R*_leaf_, hydraulic resistance of plant leaf; *R*_rest_, hydraulic resistance of rest of the plant; SPAD, relative chlorophyll content using SPAD; Tot Biom, total above-ground biomass; ΔiWUE_*g*s_, iWUE attributed to variation in *g*_s_; ΔiWUE_pc_, iWUE attributed to variation in *A*_n._

## Results

### Genotypic variation among key traits

Gas exchange variables varied among the genotypes under both watering regimes. We excluded the means for genotype R-05012-1 under WS as it responded very poorly to WS and exhibited a mean carbon assimilation rate of 1.79 µmol m^–2^ s^–1^ and stomatal conductance of 0.01 mol H_2_O m^–2^ s^–1^, which was extremely low. Mean genotype CO_2_ assimilation rate (*A*_n_) experienced a 2.2-fold variation (17.6–39.3 µmol m^–2^ s^–1^) under WW conditions and 6.1-fold variation (6.8–32.0 µmol m^–2^ s^–1^) under WS conditions (Table 3). Similarly, mean stomatal conductance (*g*_s_) experienced 2.9- (0.11–0.33 mol m^–2^ s^–1^) and 6.4-fold variation (0.01–0.16 mol m^–2^ s^–1^) under WW and WS conditions, respectively (Table 3). Operational intercellular CO_2_ concentration (*C*_i_) was similarly variable (Table 3). iWUE experienced less variation, with a fold change of 1.9 and 1.8 under WW (92–170 µmol CO_2_ mol^–1^ H_2_O) and WS (121–216 µmol CO_2_ mol^–1^ H_2_O) conditions, respectively (Table 3).

**Table 3.** Statistical summary of measured traits along with the calculated heritability and genetic variation information.

Trait	Mean	Fold change WW	Fold change WS	Genotypic variance	Treatment variance	G×T interaction variance	Residual error variance	Phenotypic variance	*H*_b_^2^	GCV (%)	PCV (%)
*A*_n_ (μmol m^–2^ s^–1^)	23.2	2.23	6.05	127.2	14 436.1	61.6	61.49	160.03	0.79	48.61	54.53
*g*_s_ (mol m^–2^ s^–1^)	0.17	2.89	6.35	0.01	1.22	0	0.01	0.01	0.75	58.82	67.92
iWUE	148.35	1.86	1.78	963	102 434	570	643.17	1281.63	0.75	20.92	24.13
*C*_i_ (μmol m^–2^ s^–1^)	111.14	2.83	6.31	2214	91 498	2170	2011.93	3339.72	0.66	42.34	52
ΦPSII	0.39	1.79	2.33	0.01	0.89	0.01	0.01	0.02	0.71	29.24	34.72
SPAD	39.48	1.92	3.21	177.8	5760.2	134.4	78.63	238.33	0.75	33.77	39.1
Ψ_midday_ (–MPa)	-1.26	2.25	2.57	0.25	46.64	0.16	0.17	0.34	0.74	39.76	46.07
Ψ_pre-dawn_ (–MPa)	-0.41	14.28	14.62	0.34	40.87	0.31	0.23	0.49	0.69	141.38	169.92
Leaf width (cm)	4.54	2.35	2.3	2.16	4	1.01	1.41	2.78	0.78	32.37	36.72
LMA (g m^–2^)	32.01	2.72	2.92	123.76	64.88	86.59	121.6	176.94	0.7	34.75	41.56
RWC (%)	80.52	1.29	1.69	69.28	2458.99	111.08	102.72	126.85	0.55	10.34	13.99
Above-ground biomass (g per plant)	21.02	15.59	15.28	209.56	2749.22	155.35	138..14	314.863	0.67	68.87	84.42

*A*_n_, carbon assimilation rate; *g*_s_, stomatal conductance; iWUE, intrinsic water use efficiency; *C*_i_, substomatal carbon dioxide concentration; ΦPSII, operating quantum yield of PSII; SPAD, chlorophyll content measured by SPAD; Ψ_midday_, midday leaf water potenial; Ψ_pre-dawn_, pre-dawn leaf water potenial; LMA, leaf mass per area; RWC, leaf relative water content; Above-ground biomass, total above-ground biomass; *H*_b_, broad-sense heritability; GCV, genetic coefficient of variation; PCV, phenotypic coefficient of variation.

*A*_n_ and *g*_s_ had higher GCV than iWUE and *C*_i_ (Table 3). All those variables exhibited high *H*_b_^2^ of ≥0.7 alongside hydraulic variables such as Ψ_midday_, apart from *C*_i_ (*H*_b_^2^=0.66) (Table 3). PCV was also similarly high (30–50%) for all those variables (Table 3), indicating that environmental factors played a role in determining variation. The genotype×treatment variance was lower than the genotype variance, indicating that most genotypes responded similarly. Final harvest parameters such as above-ground biomass also varied significantly (fold change >15), and displayed high GCV and PCV (Table 3). Mean values (with the SE) of all measured variables for every genotype under both conditions are shown in Supplementary Table S1.

### Influence of AQP-associated haplotypes on leaf intrinsic water use efficiency

We focused on the variation caused by differences between genotype groups with different AQP-associated haplotypes (see the Materials and methods). The results of this statistical analysis are presented in Table 2. Haplotypes associated with two AQPs, SbPIP1.1 and SbTIP3.2, had a significant impact on a number of key traits. For SbPIP1.1, the RP haplotype was associated with significantly higher *A*_n_ and *g*_s_ (Fig. 2A, B), while the NRP haplotype had higher iWUE (including its *g*_s_ component ΔiWUE_*g*s_) (Fig. 2C, D), LMA (Fig. 2E), and total above-ground biomass (Fig. 2F). The SbPIP1.1 NRP haplotype also had the highest ΔiWUE_pc_ of all haplotypes under WS (Supplementary Table S2; Supplementay Fig. S2H). For SbTIP3.2, The RP haplotype had higher overall *A*_n_, *g*_s_, SPAD, and ΦPSII (Fig. 2G, H, J, K, respectively), and higher Ψ_midday_ (Fig. 2L), especially under WS, while the NRP haplotype of SbTIP3.2 had higher iWUE and plant hydraulic resistance excluding the leaf (*R*_rest_) (Fig. 2I; Table 2), without an effect on biomass. The RP haplotype of SbTIP3.2 also maintained *K*_leaf_ under WS (Table 2). In summary, a common trade-off was observed between photosynthesis (*A*_n_, Φ_PSII_) and water use (*g*_s_, *K*_leaf_) for both haplotypes. Hence, genes in that chromosomal region (haplotype) probably influence those traits, including the AQP gene.

### Water stress increased iWUE, which was positively associated with above-ground biomass

**Fig. 3.**
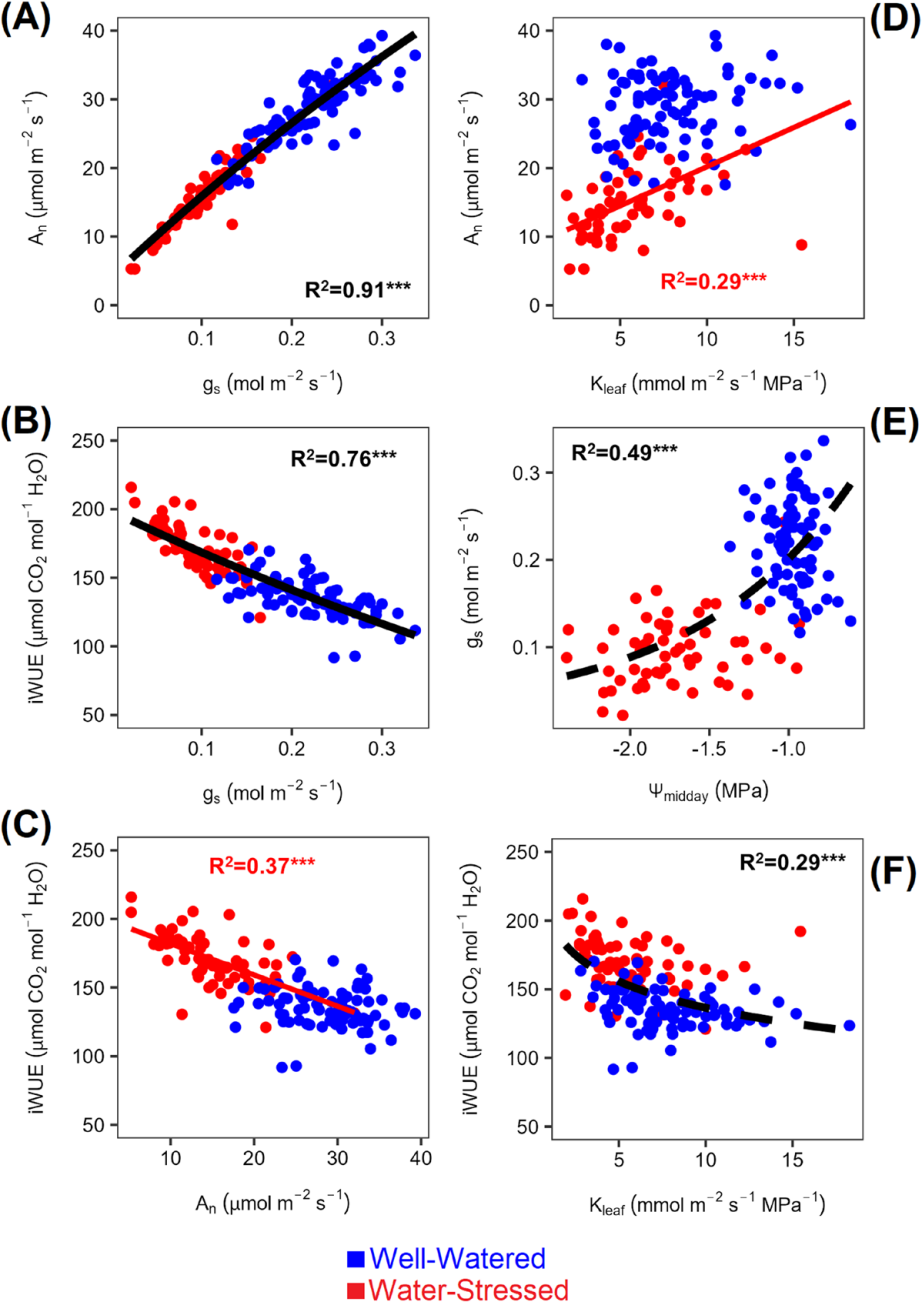
Relationship between leaf gas exchange parameters and hydraulic parameters. Data were collected on the YFEL and measured at saturating light levels (see the Materials and methods). Each point in scatter plots represents the genotype mean (*n*=3). The SE is presented in Supplementary Table S1. *R*^2^ values and related significant levels (***P<0.0001; **P<0.05) are from a Pearson product–moment correlation analysis or from the exponential fit models. Leaf water potential measurements were collected from the leaf adjacent to the YFEL used for gas exchange. (A) Net carbon assimilation rate (*A*_n_) versus stomatal conductance (*g*_s_); (B) intrinsic water use efficiency (iWUE) versus *g*_s_; (C) iWUE versus *A*_n_; (D) *A*_n_ versus leaf hydraulic conductance (*K*_leaf_); (E) *g*_s_ versus midday leaf water potential (Ψ_midday_); (F) iWUE versus *K*_leaf_.

Taking together all the genotypes, we observed that *A*_n_ and *g*_s_ correlated positively as expected (*R*^2^=0.91; *P*<0.0001; Fig. 3A), with both correlating negatively with iWUE (*R*^2^=0.92; *P*<0.0001; Fig. 3B), especially under WS for *A*_n_ (*R*=0.61; *P*<0.0001; Fig. 3C). *A*_n_ correlated positively with *K*_leaf_ under WS (*R*=0.53; *P*<0.0001; Fig. 3D), and *g*_s_ increased with higher Ψ_midday_ (*R*^2^=0.49; *P*<0.0001; Fig. 3E). Subsequently, iWUE correlated negatively with *K*_leaf_ (*R*=0.54; *P*<0.0001; Fig. 3F) as well as with more negative Ψ_midday_ and increasing *R*_rest_ and *R*_leaf_ (Supplementary Table S2). Despite this, above-ground biomass was only marginally associated with *A*_n_ when considering both WW and WS plants, but positively correlated with iWUE within each watering treatment (Supplementary Table S2). Overall the above-ground biomass production across all the genotypes under WS was regulated by a reduction in leaf area under WS (Supplementary Fig. S4D), and increasing LMA (Supplementary Table S2C).

### Components of iWUE under both well-watered and water stress conditions

**Fig. 4.**
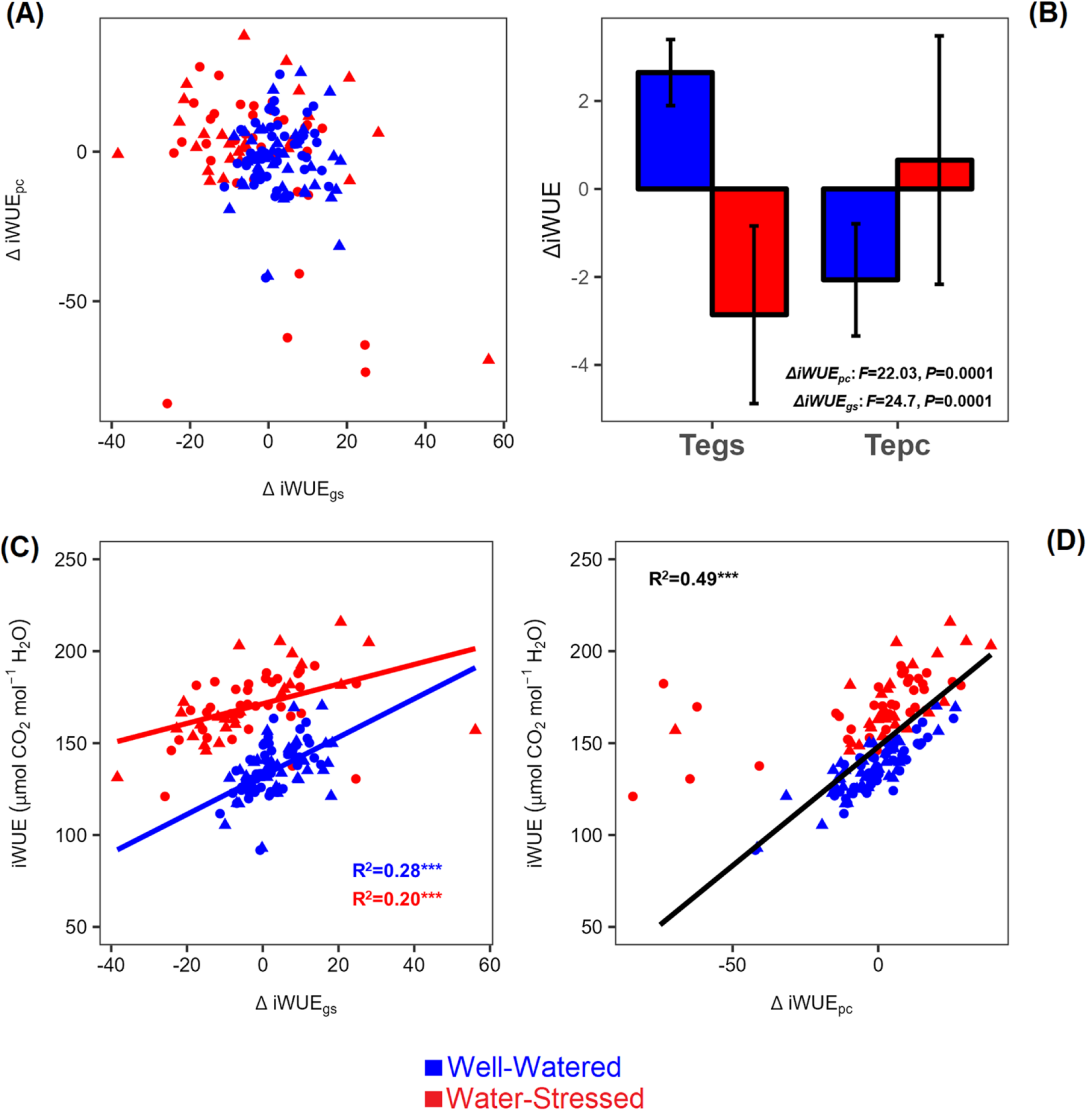
The distribution of the components of intrinsic water use efficiency (iWUE) and their relationship with each other. The values in each scatter plot compromise the mean of every genotype (*n*=3) per treatment. For the bar chart, the mean is of the genotype population (*n*=89 for WW and *n*=61 for WS). Data were collected on the YFEL and measured at saturating light levels (see the Materials and methods). Each point in scatter plots represents the genotype mean (*n*=3). The SE is presented in Supplementary Table S1. *R*^2^ values and related significant levels (***P<0.0001; **P<0.05) are from a Pearson product–moment correlation analysis or from the exponential fit models. *R*^2^ values are from a Pearson product–moment correlation analysis. (A) Variation in iWUE due to stomatal conductance (ΔiWUE_*g*s_) versus variation in iWUE due to photosynthetic capacity (ΔiWUE_pc_); (B) bar chart showing the treatment effect on ΔiWUE_*g*s_ and ΔiWUE_pc_; (C) iWUE versus ΔiWUE_*g*s_; (D) iWUE versus ΔiWUE_pc_.

**Fig. 5.**
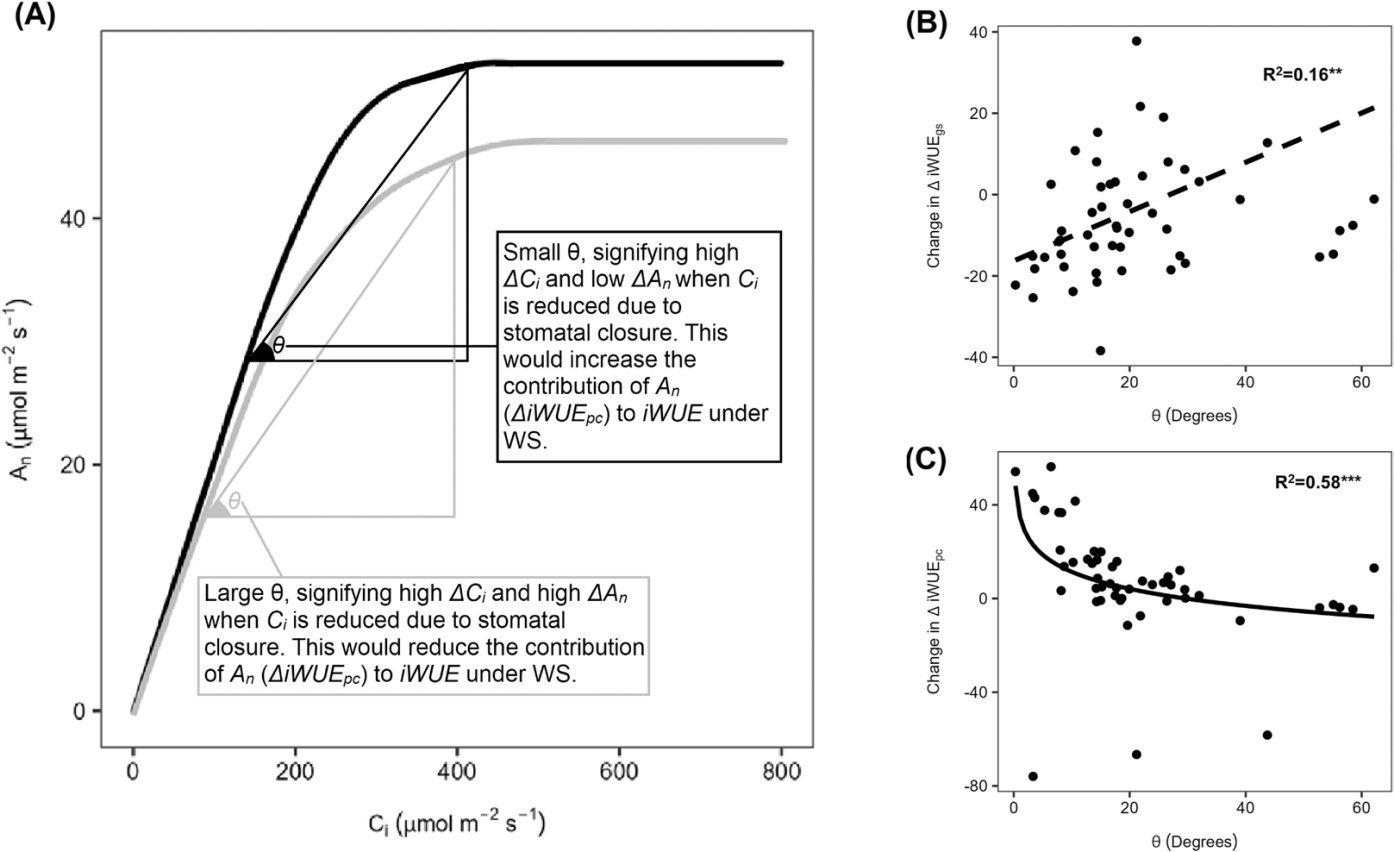
Relationship between components of intrinsic water use efficiency (iWUE) and the photosynthetic response to water limitation. (A) Conceptual representation of the change in net assimilation rate (*A*_n_) versus the operational intercellular CO_2_ concentration (*C*_i_) under progressive water stress driven mainly by stomatal limitation (black curve) or by a concomitant decrease in both stomatal and non-stomatal limitations (grey line). The figure shows potential change in *C*_i_ (Δ*C*_i_), the accompanying change in *A*_n_ (Δ*A*_n_), and the degree of change in the *A*_n_–*C*_i_ relationship [the angle θ, with θ=tan^–1^(Δ*A*_n_/Δ*C*_i_)] when the plant experiences water limitation. (B and C) Relationship between degree of change in the *A*_n_–*C*_i_ relationship (θ) from WW to WS with the change in the contribution of each component of intrinsic water use efficiency [i.e. iWUE variation due to stomatal conductance (ΔiWUE_*g*s_) and non-stomatal conductance or photosynthetic capacity (ΔiWUE_pc_)] also between WW and WS (i.e. WS–WW). Data were collected on the YFEL and measured at saturating light levels (see the Materials and methods). Each point in scatter plots represents the genotype mean (*n*=3). The SE is presented in Supplementary Table S1. *R*^2^ values and related significant levels (*** *P*<0.0001; ** *P*<0.05) are from a Pearson product–moment correlation analysis or from the exponential fit models.

We separated iWUE into a component attributed to the variation in *A*_n_ (ΔiWUE_pc_) and another attributed to variation in *g*_s_ (ΔiWUE_*g*s_) (Supplementary Fig. S2). The two components did not correlate with each other (Fig. 4A), but both positively correlated with iWUE (*R*=0.45–0.7, *P*<0.0001; Fig. 4C, D). ΔiWUE_*g*s_ was significantly higher under WW than ΔiWUE_pc_, while the opposite was true under WS (Fig. 4B). Increased iWUE associated with ΔiWUE_pc_ under WS occurs because photosynthesis decreases less than *C*_i_ (lower θ, Fig. 5A) due to the maintenance of the CCM under WS. Indeed, genotypes that increased *Δ*iWUE_pc_ under WS compared with WW had lower θ (*R*^2^=0.58; *P*<0.0001; Fig. 5C), while genotypes that increased their ΔiWUE_*g*s_ under WS showed a weak association with increasing θ (*R*=0.4; *P*<0.05; Fig. 5B). Hence, genotypes that maintained photosynthetic capacity under low *C*_i_ can combine iWUE with photosynthetic performance under WS.

**Fig. 6.**
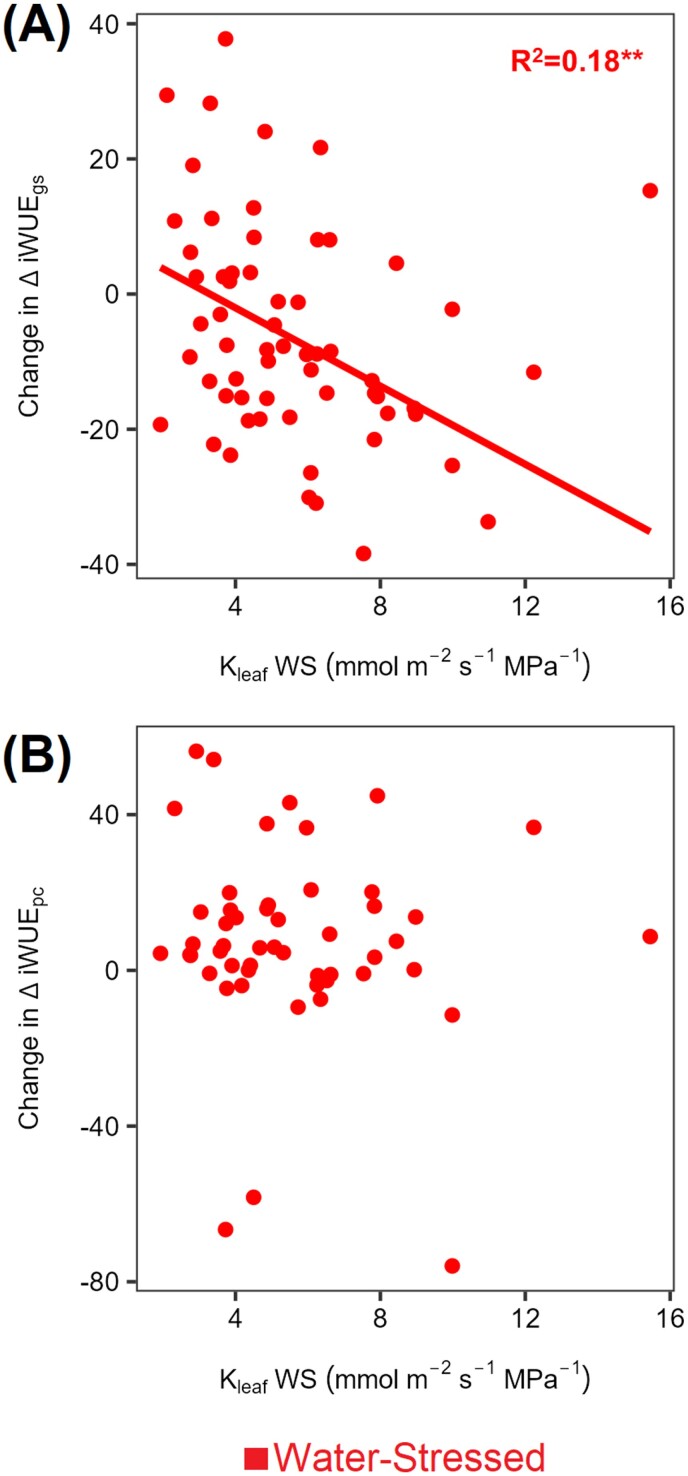
Relationship between the change in the contribution of each component of intrinsic water use efficiency from WW to WS (i.e. WS–WW) with leaf hydraulic conductance (*K*_leaf_) under WS. Data were collected on the YFEL and measured at saturating light levels (see the Materials and methods). Each point in scatter plots represents the genotype mean (*n*=3). The SE is presented in Supplementary Table S1. *R*^2^ values and related significant levels (***P<0.0001; **P<0.05) are from a Pearson product–moment correlation analysis or from the exponential fit models. Leaf water potential measurements were collected from the leaf adjacent to the YFEL used for gas exchange. (A) Change in iWUE variation due to stomatal conductance (ΔiWUE_*g*s_) versus *K*_leaf_ WS; (B) change in iWUE variation due to photosynthetic capacity (ΔiWUE_pc_) versus *K*_leaf_ WS.

We examined the link between increased ΔiWUE_pc_ under WS and a better hydraulic response. No correlation was found between the increase in ΔiWUE_pc_ under WS and higher *K*_leaf_ (Fig. 6B). Instead, increasing ΔiWUE_*g*s_ was associated with lower *K*_leaf_ (*R*=0.43; *P*<0.05; Fig. 6A) and more negative Ψ_midday_ (Supplementary Fig. S5C), but this did not apply to ΔiWUE_pc_ (Supplementary Fig. S5D).

## Discussion

This study screened a large number of sorghum genotypes that shared most of their genetic composition but differed in key gene blocks (haplotypes) that are associated with certain AQP genes inherited from the elite or exotic parental lines. This population was used to test for genetic variation in the response of iWUE and its components to WS, and their relationship with productivity and plant hydraulics. Our key findings were: (i) there was significant diversity in many variables related to productivity which also presented high broad-sense heritability; (ii) some of this diversity is underpinned by differences in haplotypes associated with some AQPs especially for gas exchange and hydraulic parameters; (iii) the non-stomatal component of iWUE (ΔiWUE_pc_) was associated with higher iWUE under both WW and WS conditions; and (iv) genotypes with higher ΔiWUE_pc_ were not sensitive to low *K*_leaf_ under WS. We discuss those findings below.

### Breeding for high iWUE and possible impact of SbAQPs

Breeding for high iWUE in C_4_ crops, and particularly in sorghum, has been discouraged due to lack of sufficient variation among genotypes reported in earlier studies, lack of traits that could be easily measured in large-scale screens, and the complex physiology of iWUE, where its components such as *g*_s_ can be easily influenced by environmental factors such as VPD or WS (Condon *et al.*, 2004; Sinclair *et al.*, 2005). High *H*_b_^2^ of key parameters such as *A*_n_, *g*_s_, iWUE, LW, and SPAD under environmental variation within inbred sorghum genotypes is a significant finding [Table 3; see similar high *H*_b_^2^ in other C_4_ crops (Basnayake *et al.*, 2015; Jackson *et al.*, 2016; Li *et al.*, 2017; Ferguson *et al.*, 2023, Preprint)], considering: (i) the genotypes shared 75% of their genetic material (Fig. 1); (ii) later attempts at finding variation in iWUE were not always promising (Hammer *et al.*, 1997; Blum, 2009; Leakey *et al.*, 2019; Pan *et al.*, 2022; Zhi *et al.*, 2022; Al-Salman *et al.*, 2023); and (iii) previous key improvements in sorghum, such as the stay-green trait, were achieved via a significant breeding contribution from wild sorghum relatives (Ochieng *et al.*, 2021). Despite the high *H*_b_^2^, the high PCV of iWUE (Table 3) meant that environmental factors that affect *g*_s_ played an important role in driving variation of iWUE.

Hence, success in breeding for high iWUE is dependent on understanding the effect of different adaptive traits on iWUE and, vice versa, under different environments (Reynolds *et al.*, 1994; Araus *et al.*, 2003). To screen for and expand the suite of such adaptive traits, we partitioned iWUE into a non-stomatal component (ΔiWUE_pc_) and a stomatal component (ΔiWUE_*g*s_) (as in Gilbert *et al.*, 2011 and Li *et al.*, 2017), allowing us to reconcile high iWUE with photosynthetic performance and to link iWUE components to traits such as *K*_leaf_ or θ. Also, the variation in iWUE we found was associated with haplotypes where specific AQP genes were positively ascribed to parental lines from contrasting geographical regions and climates. This genetic information may be used for further specific studies addressing the role of such AQPs, or the accompanying genes, in sorghum performances under both WW and WS conditions.

Our results hint at a possible role for two AQPs (SbPIP1.1 and SbTIP3.2) that might influence iWUE and related traits (Fig. 2). AQPs change the permeability of cell membranes, facilitating water transport from the apoplastic region to the inner cells, and vice versa from the xylem to the stomata in the leaves, and hence keeping leaf cells hydrated during transpiration (Shope *et al.*, 2008; Mott and Peak, 2010; Chaumont and Tyerman, 2014; Li *et al.*, 2014). Water needs to enter guard cells for stomatal opening and increasing *g*_s_ (Franks and Farquhar, 2007; Rockwell *et al.*, 2014; Buckley *et al.*, 2017), which subsequently increases *A*_n_ and reduces iWUE (Fig. 2). The ability to maintain higher *g*_s_ can be related to improved leaf hydraulic traits (Brodribb *et al.*, 2005). For example, the RP SbTIP3.2 haplotype had higher Ψ_midday_, higher *g*_s_, and maintained *K*_leaf_ under WS compared with the NRP (Fig. 2L; Table 2; Supplementary Table S3). TIP AQPs are localized in the vacuolar membrane (tonoplast) and play a key role in maintaining cell turgor, possibly explaining the effect on leaf water status of SbTIP3.2 (Chaumont and Tyerman, 2014). Ectopic expression of a TIP gene has demonstrated that increased AQP activity generally leads to anisohydric behaviour by promoting water transport within the plant and preventing stomatal closure (Maurel *et al.*, 2015). Furthermore, TIPs and PIP2s are known to transport the most abundant reactive oxygen species (H_2_O_2_), which may have a role in plant cell signalling and even in detoxication of reactive oxygen species (Maurel *et al.*, 2015). However, WS also alters leaf pH and triggers abscisic acid (ABA) production and transport, which impact the activity of proton pumps associated with AQP activation and probably reducing AQP expression levels (Alexandersson *et al.*, 2005; Miyazawa *et al.*, 2008; Shatil-Cohen *et al.*, 2011; Pantin *et al.*, 2013; Shivaraj *et al.*, 2021). Therefore, it is also likely that other genes within that haplotype contribute to this response. Increased *A*_n_ in RP SbTIP3.2 may be attributed to the higher *g*_s_, but also to more efficient reactive oxygen species-scavenging systems, which is in agreement with their higher chlorophyll content (as surrogated by SPAD) and electron transport rate, as inferred by higher ΦPSII (Fig. 2J, K). We did find significant differences in those two parameters between the RP and NRP haplotypes associated with SbTIP4.3/4.4 but with no impact on *A*_n_ (Table 2). Given that SbTIP4.3/4.4 genes are located in chromosome 3, but SbTIP 3.2 and SbPIP 1.1 are in chromosome 6 close to each other (Reddy *et al.*, 2015), and that both haplotypes from the elite parental line used in the Australian breeding programme (RP SbTIP 3.2 and RP SbPIP 1.1) had higher *A*_n_ and *g*_s_, although lower iWUE than NRP haplotypes, they can be exploited to increase *A*_n_ under predominantly WW conditions.

However, the NRP haplotype (associated with the parental line IS9710 originated from the dry region of Sudan) of the AQP SbPIP 1.1 had significantly higher ΔiWUE_*g*s_, iWUE, above-ground biomass, and LMA than the RP SbPIP 1.1 haplotype under WW conditions, suggesting a trade-off between higher carbon assimilation by unit of leaf area of the RP Australian line, but total plant assimilation of the NRP Sudanese line. This same haplotype (NRP SbPIP1.1) had the highest ΔiWUE_pc_ of all haplotypes under WS, but also the highest above-ground biomass and highest iWUE of all haplotypes under WS, suggesting a probable function of SbPIP1.1 from the Sudanese haplotype also in the WS response. Further studies are required to ascertain the functions of SbAQPs genes, and related genes associated with the haplotypes identified in this study, and the precise role of the highlighted AQPs in abiotic stress responses.

### Screening for both high *A*_n_ and iWUE under water stress may be achieved through *C*_i_ and might be associated with above-ground plant biomass

In C_4_ plants, increased *g*_s_ under WW conditions may not be advantageous because C_4_ photosynthesis saturates close to their operational *C*_i_, resulting in the strong dependence of iWUE on *g*_s_ (Figs 3B, 4B) as observed in previous studies (Jackson *et al.*, 2016; Cano *et al.*, 2019; Pignon *et al.*, 2021b; Pan *et al.*, 2022; Al-Salman *et al.*, 2023). Under WS, lower *g*_s_ increases iWUE overall but also imposes a diffusional limitation on *A*_n_ by lowering *C*_i_. Hence, variation in photosynthetic capacity can overcome this diffusional limitation and increase iWUE by maximizing *A*_n_ for a given *g*_s_ (Fig. 4B), or rather *C*_i_ as shown for genotypes with higher ΔiWUE_pc_ having smaller *A*_n_ reductions compared with *C*_i_ (Fig. 5C) (Collyer and Adams, 2007; Gilbert *et al.*, 2011; Li *et al.*, 2017). *C*_i_ can then be an indicator of not just iWUE, but of ΔiWUE_pc_ (see strong association of *C*_i_ with *Δ*iWUE_pc_ compared with ΔiWUE_*g*s_ in Supplementary Table S2), confirming previous assumptions about *C*_i_ as an integrator of iWUE and productivity in C_4_ plants (Ghannoum, 2016; Jackson *et al.*, 2016; Condon, 2020). However, we found no strong relationship between *A*_n_ or ΔiWUE_pc_ and biomass, apart from a weak relationship between *A*_n_ and panicle size when both treatments are grouped (Supplementary Table S2A). We also detected a weak (*R*=0.27) but statistically significant relationship between iWUE and total biomass (Supplementary Table S1B, C). We note here that the significant, but low *R*^2^ (and *R*) values displayed in our data are typical of studies focused on intra-specific diversity especially within crops and especially when exploring complex physiological traits that are underpinned by several processes (Pignon *et al.*, 2021a; Li *et al.*, 2022; Zhi *et al.*, 2022).

Efficient use of water at the leaf scale [higher leaf Ψ_midday_ and lower plant hydraulic resistance (*R*_rest_) (Supplementary Table S2B, C)] combined with morphological adaptations such as narrower leaves (Supplementary Fig. S4A) and higher leaf density [as LMA increased but leaf thickness only marginally reduced under WS (Supplementary Fig. S4C, D)] can lead to reduced *g*_s_ [see positive association between *g*_s_ and LW in Supplementary Table S2 also found in Pan *et al.* (2022) and Al-Salman *et al.* (2023)]. This results in reduced water use and high iWUE, leading to water conservation in the soil for biomass accumulation later in the season (Seneweera *et al.*, 2001; Vadez, 2019). Previous work on stay-green sorghum (most of our population is stay-green) showed that plant water use is lower during vegetative and early-reproductive stage, which is when we measured gas exchange, before ramping up during grain filling (Borrell *et al.*, 1999, [Bibr CIT0009][Bibr CIT0009], b, George-Jaeggli *et al.*, 2017). There is still scepticism about how much iWUE or photosynthesis *per se* can help drive productivity in future environments (Sinclair, 2012; Sinclair *et al.*, 2019), especially in C_4_ crops (Sales *et al.*, 2021), since the yield of grain crops is heavily influenced by changing source–sink relationships and seasonal timings (Dingkuhn *et al.*, 2020; Fabre *et al.*, 2020). The impact of leaf-level physiological traits on whole-plant productivity under different conditions requires a comprehensive approach (Sreeman *et al.*, 2018; Tardieu *et al.*, 2018).

### Road map to select promising sorghum genotypes under soil water deficit

A comprehensive physiological approach to crop drought response requires understanding of the relevant traits in response to the specific environment (Tardieu *et al.*, 2018). Too high iWUE under soil water deficit due to lowering *g*_s_ is not desirable because this indicates that the plant is experiencing moderate to severe WS and has an overall lower plant water status and reduced *K*_leaf_ (Blum, 2009; Sinclair, 2012, 2018). Indeed, reductions of *K*_leaf_ and Ψ_midday_ were associated with increasing ΔiWUE_*g*s_ (and more closed stomata) [Fig. 6A; Supplementary Fig. S5C; Supplementary Table S2, coming at the expense of photosynthesis (see negative correlation between ΦPSII and ΔiWUE_*g*s_ under WS (Supplementary Fig. S5A)]. Higher *K*_leaf_ can help maintain *A*_n_ under low *C*_i_. Selecting for genotypes that respond to soil drought by taking some hydraulic ‘risks’ (maintaining *K*_leaf_) and keeping stomata relatively open under increasing WS may increase iWUE by increasing carbon accumulation as seen already in some grasses (Holloway-Phillips and Brodribb, 2011). Such a genotype would operate where the minimum *g*_s_ is attained for the maximum *A*_n_ (hence, high iWUE associated with high ΔiWUE_pc_) (Fig. 5). Traits that enable ‘risky’ hydraulic behaviour without risk of cavitation can include deeper and more conductive roots, wider xylem vessels (Scoffoni *et al.*, 2011), and higher leaf vein density [already associated with higher iWUE in sorghum (Pan *et al.*, 2022; Al-Salman *et al.*, 2023)]. Other important traits can be related to extra-xylem conductivities such as enhanced mesophyll conductance (of CO_2_ or H_2_O), reduced bundle sheath conductance, reduced airspace, and more compact mesophyll structure around veins (Buckley, 2015; Buckley *et al.*, 2015; Sack *et al.*, 2015; Fiorin *et al.*, 2016; Xiong *et al.*, 2017, 2018; Pathare *et al.*, 2020; Al-Salman *et al.*, 2023), which are all processes influenced by AQPs (Maurel *et al.*, 2015; Negin and Moshelion, 2016; Groszmann *et al.*, 2017; Ermakova *et al.*, 2021). Combining water use strategy with gas exchange mechanisms is crucial to clarifying the benefits of increasing iWUE under different conditions (Liang *et al.*, 2023).

### Conclusion

We conducted a physiologically extensive screen of >80 sorghum genotypes selected based on differences in haplotypes originating from different parents from different origins and climates. We found significant variation among key traits, with some underpinned by differences between AQP-associated haplotypes inherited from an elite and exotic parent, providing possible target genomic regions for beneficial traits. Partitioning the components of iWUE into stomatal and non-stomatal components of *A*_n_ allowed us to find a physiological mechanism that can lead to attainment of high iWUE without hindering photosynthesis or drought tolerance. We explained this mechanism through the connection between leaf and plant hydraulic conductivities and the maintenance of assimilation rates under low *C*_i_. These findings provide a possible roadmap to expand the range of traits linked to iWUE in C_4_ crops, offer possible avenues to bridge the trade-off between iWUE and productivity, and strengthen the case for AQPs as possible key players in this endeavour.

## Supplementary data

The following supplementary data are available at *JXB* online.

Table S1. Variable means with SE for each genotype at each treatment.

Table S2. Pearson correlation matrix of all the variables.

Table S3. Means for all traits for each RP/NRP AQP haplotype group.

Table S4. ANOVA comparison of parameters between chambers.

Fig. S1. Average diurnal glasshouse conditions.

Fig. S2. Calculation of different iWUE components

Fig. S3. Correlation between iWUE components calculated based on a global reference curve and a genotype reference.

Fig. S4. Boxplots of plant morphological parameter distributions across the two treatments.

Fig. S5. Relationship between components of iWUE and leaf water potential and photosynthetic efficiency.

Fig. S6. Bar charts showing the effects of water stress on iWUE components in different haplotypes.

Fig. S7. Relationship between carbon assimilation, chlorophyll content, and efficiency of PSII.

Fig. S8. Relationship between the change in the carbon assimilation–intercellular CO_2_ concentration relationship and components of iWUE.

Fig. S9. Relationship between change in components of iWUE and change in hydraulic conductivity.

Fig. S10. Relationships between hydraulic conductivity and water potentials.

Fig. S11. Boxplots of gas exchange parameter distributions across the two treatments.

Fig. S12. Boxplots of hydraulic parameter distributions across the two treatments.

erae418_suppl_Supplementary_Figures_S1-S5_Tables_S2-S3

erae418_suppl_Supplementary_Tables_S1

## Data Availability

The data generated and analysed for this study are available from the corresponding author on request.
