# Supplementary material for: High water use efficiency due to maintenance of photosynthetic capacity in sorghum under water stress
Source: J Exp Bot. 2024 Oct 8;75(21):6778–95. doi: 10.1093/jxb/erae418 (PMC11565205; doi:10.1093/jxb/erae418)
Supplement: erae418_suppl_Supplementary_Figures_S1-S5_Tables_S2-S3 [file erae418_suppl_supplementary_figures_s1-s5_tables_s2-s3.pdf]

Tables S1A and S1B, showing the genotype mean for the measured traits with standard error, are in their own excel file because of their size.

Table S2A. Pearson product moment correlation analysis of the global relationships (across the two treatments) between the measured variables, using genotype means (*n*=3). (***: *P*<0.0001; **:*P*<0.05).

	g_s	*iWUE*	$\Delta iWUE_{pc}$	$\Delta iWUE_{gs}$	C_i	*Φ PSII*	$\Psi_{pre\text{-}dawn}$	Ψ_{midday}	K_{plant}	K_{leaf}	R_{leaf}	R_{rest}	*LW*	*LT*	*TBiom*	*PS*	*SPAD*
A_n (μmol m^{-2} s^{-1})	0.94***	-0.59***	0.17***	-0.54***	0.34***	0.52***	0.51***	0.49***	0.44***	0.38***	-0.51***	-0.55***	0.26***	0.1**	0.14**	0.22***	0.54***
g_s (mol m^{-2} s^{-1})	-	-0.76***	ns	-0.51***	0.55***	0.5***	0.49***	0.48***	0.49****	0.42***	-0.54***	-0.56***	0.18***	0.11**	0.11**	0.16**	0.46***
iWUE	-	-	0.69***	0.33***	-0.93***	-0.42***	-0.53***	-0.58***	-0.43***	-0.36***	0.53***	0.57***	0.15**	ns	ns	ns	-0.24***
$\Delta iWUE_{pc}$	-	-	-	-0.22***	-0.74***	ns	ns	ns	ns	ns	ns	ns	0.26***	ns	ns	ns	0.16***
$\Delta iWUE_{gs}$	-	-	-	-	-0.23***	-0.16**	-0.24***	-0.15**	-0.25***	-0.29***	ns	ns	ns	ns	ns	ns	-0.15**
C_i *(ppm)*	-	-	-	-	-	0.21***	0.35***	0.45***	0.28***	0.21***	-0.32***	-0.45***	-0.28***	ns	-0.1**	-0.11**	ns
ΦPSII	-	-	-	-	-	-	0.5***	0.45***	0.23***	0.18***	-0.42***	-0.47***	0.11**	0.34***	0.12**	0.16**	0.51***
$\Psi_{pre\text{-}dawn}$ (-MPa)	-	-	-	-	-	-	-	0.72***	0.12**	0.23***	-0.48***	-0.36***	-0.17***	ns	ns	ns	0.34***
Ψ_{midday} (-MPa)	-	-	-	-	-	-	-	-	0.48***	0.33***	-0.59***	-0.65***	-0.27***	0.1**	ns	ns	0.22***
K_{plant} (mmol m^{-2} s^{-1} MPa^{-1})	-	-	-	-	-	-	-	-	-	0.61***	-0.46***	-0.56***	ns	ns	ns	ns	ns
K_{leaf} (mmol m^{-2} s^{-1} MPa^{-1})	-	-	-	-	-	-	-	-	-	-	-0.62***	-0.26**	ns	ns	ns	ns	ns
R_{leaf}	-	-	-	-	-	-	-	-	-	-	-	0.35***	ns	-0.18**	ns	ns	-0.23**
R_{rest}	-	-	-	-	-	-	-	-	-	-	-	-	-0.18**	-0.24**	ns	ns	-0.34***
Leaf Width (cm)	-	-	-	-	-	-	-	-	-	-	-	-	-	0.1**	0.42***	0.26***	0.29***
Leaf Thickness (mm)	-	-	-	-	-	-	-	-	-	-	-	-	-	-	0.11**	ns	0.13**
Total Biomass	-	-	-	-	-	-	-	-	-	-	-	-	-	-	-	0.42***	0.16***
Panicle size	-	-	-	-	-	-	-	-	-	-	-	-	-	-	-	-	0.27***

A_n: Carbon assimilation rate; g_s: Stomatal conductance; *iWUE*: Instantaneous water use efficiency; $\Delta iWUE_{pc}$: *iWUE* attributed to variation in A_n; $\Delta iWUE_{gs}$: *iWUE* attributed to variation in g_s; C_i: Sub-stomatal carbon dioxide concentration; *ΦPSII*: Operating quantum yield of photosystem II; $\Psi_{pre\text{-}dawn}$ and Ψ_{midday}: Midday leaf water potenial; K_{plant}: Plant hydraulic conductivity; K_{leaf}: Leaf hydraulic conductivity; R_{leaf}: Hydraulic resistance of the leaf; R_{rest}: Hydraulic resistance of non-leaf parts of the plant; *LW*: Leaf width; *LT*: Leaf thickness; *TBiom:* Total aboveground biomass; *LMA*: Leaf mass per area; *SPAD*: Relative Chlorophyll content based on SPAD.

Table S2B. Pearson product moment correlation analysis of the relationships between the measured variables in the *well-watered* treatment, using genotype means (*n*=3). (***: *P*<0.0001; **:*P*<0.05).

	g_s	*iWUE*	$\Delta iWUE_{pc}$	$\Delta iWUE_{gs}$	C_i	*Φ PSII*	$\Psi_{pre\text{-}dawn}$	Ψ_{midday}	K_{plant}	K_{leaf}	R_{leaf}	R_{rest}	*LW*	*LT*	*LMA*	*TBiom*	*SPAD*	*θ*
A_n (μmol m^{-2} s^{-1})	0.86***	ns	0.35***	-0.88***	ns	ns	ns	ns	ns	ns	ns	ns	0.36***	ns	ns	ns	0.35***	ns
g_s (mol m^{-2} s^{-1})	-	-0.6***	ns	-0.92***	0.3**	ns	ns	ns	ns	0.25**	-0.28**	-0.23**	0.18***	0.11**	ns	0.11**	0.46***	ns
iWUE	-	-	0.85***	0.82***	-0.9***	ns	ns	ns	-0.25**	-0.28**	0.38**	0.31**	ns	ns	0.23**	0.25**	0.224**	ns
$\Delta iWUE_{pc}$	-	-	-	ns	-0.91***	ns	ns	ns	-0.22**	ns	0.24**	ns	0.41***	ns	0.23**	0.3**	0.37***	ns
$\Delta iWUE_{gs}$	-	-	-	-	-0.23**	ns	-0.2**	ns	ns	-0.23**	0.32**	0.24**	-0.29**	ns	ns	ns	ns	ns
C_i *(ppm)*	-	-	-	-	-	ns	ns	0.22**	0.31**	0.34**	-0.41***	ns	-0.32**	ns	-0.22**	-0.1**	-0.37**	ns
ΦPSII	-	-	-	-	-	-	ns	ns	ns	ns	ns	ns	ns	0.39***	ns	ns	0.38***	ns
$\Psi_{pre\text{-}dawn}$ (-MPa)	-	-	-	-	-	-	-	ns	-0.56***	ns	ns	0.22**	ns	ns	ns	ns	0.32**	ns
Ψ_{midday} (-MPa)	-	-	-	-	-	-	-	-	0.46***	0.47***	-0.52***	-0.29**	-0.21**	ns	ns	ns	-0.26**	ns
K_{plant} (mmol m^{-2} s^{-1} MPa^{-1})	-	-	-	-	-	-	-	-	-	0.71***	-0.54**	-0.65**	-0.21**	ns	ns	ns	-0.26**	ns
K_{leaf} (mmol m^{-2} s^{-1} MPa^{-1})	-	-	-	-	-	-	-	-	-	-	-0.7***	-0.43***	ns	ns	ns	ns	ns	ns
R_{leaf}	-	-	-	-	-	-	-	-	-	-	-	0.36***	ns	ns	ns	ns	0.22**	ns
R_{rest}	-	-	-	-	-	-	-	-	-	-	-	-	ns	-0.23**	ns	ns	ns	ns
Leaf Width (cm)	-	-	-	-	-	-	-	-	-	-	-	-	-		ns	ns	ns	ns
Leaf Thickness (mm)	-	-	-	-	-	-	-	-	-	-	-	-	-	-	ns	ns	ns	ns
LMA	-	-	-	-	-	-	-	-	-	-	-	-	-	-	-	0.29**	0.3**	-0.34**
Total Biomass	-	-	-	-	-	-	-	-	-	-	-	-	-	-	-	-	ns	ns
SPAD	-	-	-	-	-	-	-	-	-	-	-	-	-	-	-	-	-	ns

A_n: Carbon assimilation rate; g_s: Stomatal conductance; *iWUE*: Instantaneous water use efficiency; $\Delta iWUE_{pc}$: *iWUE* attributed to variation in A_n; $\Delta iWUE_{gs}$: *iWUE* attributed to variation in g_s; C_i: Sub-stomatal carbon dioxide concentration; *ΦPSII*: Operating quantum yield of photosystem II; $\Psi_{pre\text{-}dawn}$ and Ψ_{midday}: Midday leaf water potenial; K_{plant}: Plant hydraulic conductivity; K_{leaf}: Leaf hydraulic conductivity; R_{leaf}: Hydraulic resistance of the leaf; R_{rest}: Hydraulic resistance of non-leaf parts of the plant; *LW*: Leaf width; *LT*: Leaf thickness; *TBiom:* Total aboeground biomass; *LMA*: Leaf mass per area; *SPAD*: Relative Chlorophyll content based on SPAD; *θ*: $\tan^{-1}((An\,WW - An\,WS)/(Ci\,WW - Ci\,WS))$.

Table S2C. Pearson product moment correlation analysis of the relationships between the measured variables in the *water-stressed* treatment, using genotype means (*n*=3). (***: *P*<0.0001; **:*P*<0.05).

	g_s	*iWUE*	$\Delta iWUE_{pc}$	$\Delta iWUE_{gs}$	C_i	*Φ PSII*	$\Psi_{pre\text{-}dawn}$	Ψ_{midday}	K_{plant}	K_{leaf}	R_{leaf}	R_{rest}	*LW*	*LT*	*LMA*	*TBiom*	*SPAD*	*θ*
A_n (μmol m^{-2} s^{-1})	0.94***	-0.61**	ns	-0.89***	ns	0.26**	ns	ns	ns	0.38**	-0.36**	ns	ns	-0.36**	ns	ns	ns	ns
g_s (mol m^{-2} s^{-1})	-	-0.77***	ns	-0.91***	ns	0.28**	ns	0.27**	0.28**	0.37**	-0.37**	ns	ns	-0.32**	ns	ns	ns	ns
iWUE	-	-	0.56***	0.45***	-0.55***	ns	ns	-0.36**	-0.32**	-0.27**	0.27**	0.29**	0.37**	0.35**	0.12**	0.27**	ns	-27**
$\Delta iWUE_{pc}$	-	-	-	-0.33**	-0.53**	ns	ns	ns	ns	ns	ns	ns	ns	ns	ns	ns	ns	ns
$\Delta iWUE_{gs}$	-	-	-	-	ns	-0.32**	-0.37**	-0.33**	ns	-0.3**	0.31**	ns	ns	ns	ns	ns	ns	ns
C_i (ppm)	-	-	-	-	-	ns	ns	0.28**	ns	ns	ns	ns	-0.39**	ns	ns	ns	ns	ns
ΦPSII	-	-	-	-	-	-	0.58***	0.31**	ns	ns	ns	ns	ns	ns	ns	ns	0.5***	ns
$\Psi_{pre\text{-}dawn}$ (-MPa)	-	-	-	-	-	-	-	0.41***	-0.29**	ns	ns	ns	-0.36**	ns	ns	ns	ns	ns
Ψ_{midday} (-MPa)	-	-	-	-	-	-	-	-	0.31**	ns	-0.36***	-0.37**	-0.54***	ns	ns	-0.37**	ns	ns
K_{plant} (mmol m^{-2} s^{-1} MPa^{-1})	-	-	-	-	-	-	-	-	-	ns	ns	-0.51***	ns	ns	ns	ns	ns	ns
K_{leaf} (mmol m^{-2} s^{-1} MPa^{-1})	-	-	-	-	-	-	-	-	-	-	-0.5***	ns	ns	-0.45***	ns	ns	ns	ns
R_{leaf}	-	-	-	-	-	-	-	-	-	-	-	ns	ns	ns	ns	ns	ns	ns
R_{rest}	-	-	-	-	-	-	-	-	-	-	-	-	ns	ns	ns	ns	ns	0.32**
Leaf Width (cm)	-	-	-	-	-	-	-	-	-	-	-	-	-	ns	ns	ns	ns	ns
Leaf Thickness (mm)	-	-	-	-	-	-	-	-	-	-	-	-	-	-	ns	ns	ns	ns
LMA	-	-	-	-	-	-	-	-	-	-	-	-	-	-	-	0.54***	ns	ns
Total Biomass	-	-	-	-	-	-	-	-	-	-	-	-	-	-	-	-	ns	ns
SPAD	-	-	-	-	-	-	-	-	-	-	-	-	-	-	-	-	-	ns

A_n: Carbon assimilation rate; g_s: Stomatal conductance; *iWUE*: Instantaneous water use efficiency; $\Delta iWUE_{pc}$: *iWUE* attributed to variation in A_n; $\Delta iWUE_{gs}$: *iWUE* attributed to variation in g_s; C_i: Sub-stomatal carbon dioxide concentration; *ΦPSII*: Operating quantum yield of photosystem II; $\Psi_{pre\text{-}dawn}$ and Ψ_{midday}: Midday leaf water potenial; K_{plant}: Plant hydraulic conductivity; K_{leaf}: Leaf hydraulic conductivity; R_{leaf}: Hydraulic resistance of the leaf; R_{rest}: Hydraulic resistance of non-leaf parts of the plant; *LW*: Leaf width; *LT*: Leaf thickness; *TBiom:* Total aboeground biomass; *LMA*: Leaf mass per area; *SPAD*: Relative Chlorophyll content based on SPAD; *θ*: $\tan^{-1}((An\,WW - An\,WS)/(Ci\,WW\text{-}Ci\,WS))$.

Table S3. Means of the traits for each RP/NRP population representing the specific aquaporin haplotype with standard error. *n is* for the number of independent genotypes per haplotype group for that specific aquaporin for that treatment.

Exotic Parent	Aquaporin	Population & Treatment	A_n	g_s	iWUE	ΦPSII	Ψ_{leaf}	K_{leaf}	LMA	R_{leaf}	R_{rest}	SPAD	Tot Biom	$\Delta iWUE_{gs}$	$\Delta iWUE_{pc}$
**FF_RTx7000**	**PIP 2.7**	**RP - WW (*n* =6)**	27.89 (2.25)	0.22 (0.03)	132.67 (5.77)	0.41 (0.02)	-0.89 (0.05)	8.47 (0.96)	31.07 (2.54)	0.14 (0.01)	0.1 (0.02)	42.5 (2.39)	15.52 (2.21)	3.05 (3.08)	-5.04 (4.59)
		**RP - WS (*n* =3)**	17.76 (2.3)	0.11 (0.02)	165.46 (6.86)	0.4 (0.01)	-1.33 (0.17)	8.24 (2.59)	27.9 (3.47)	0.23 (0.05)	0.26 (0.07)	34.19 (1.63)	11.98 (1.5)	-9.8 (7.47)	4.69 (6.28)
		**NRP - WW (*n* =7)**	24.82 (2.36)	0.2 (0.02)	128.64 (4.59)	0.4 (0.02)	-0.86 (0.04)	7.43 (0.75)	29.55 (2.6)	0.15 (0.01)	0.06 (0.02)	38 (2)	13.05 (2.43)	7.14 (3.47)	-13.15 (5.47)
		**NRP - WS (*n* =4)**	17 (3.23)	0.12 (0.03)	166.45 (11.02)	0.39 (0.03)	-1.52 (0.19)	5.54 (0.92)	23.99 (2.3)	0.27 (0.06)	0.65 (0.24)	34.58 (3.72)	10.69 (1.37)	-8 (8.31)	3.88 (4.44)
**QL12**	**TIP 1.1**	**RP - WW (*n* =14)**	29.15 (4.61)	0.23 (0.04)	135.02 (21.35)	0.45 (0.07)	-0.97 (-0.15)	8.07 (1.28)	33.18 (5.25)	0.18 (0.03)	0.1 (0.02)	45.31 (7.16)	20.4 (3.23)	2.12 (0.34)	-1.76 (-0.28)
		**RP - WS (*n* =11)**	13.33 (2.91)	0.08 (0.02)	171.74 (37.48)	0.32 (0.07)	-1.93 (-0.42)	4.71 (1.03)	31.29 (6.83)	0.33 (0.07)	0.55 (0.12)	35.09 (7.66)	18.61 (4.06)	1.4 (0.31)	-6.36 (-1.39)
		**NRP - WW (*n* =6)**	31.33 (7.01)	0.26 (0.06)	130.12 (29.1)	0.46 (0.1)	-1.01 (-0.23)	6.78 (1.52)	35.47 (7.93)	0.16 (0.04)	0.1 (0.02)	47.42 (10.6)	24.68 (5.52)	-0.41 (-0.09)	-0.69 (-0.15)
		**NRP - WS (*n* =4)**	14.63 (4.63)	0.09 (0.03)	168.73 (53.36)	0.33 (0.11)	-1.74 (-0.55)	6.09 (1.93)	32.97 (10.43)	0.3 (0.09)	0.68 (0.21)	38.28 (12.11)	20.62 (6.52)	-1.05 (-0.33)	-14.85 (-4.7)
**QL12**	**TIP 3.2**	**RP - WW (*n* =13)**	30.65 (1.25)	0.25 (0.02)	133.85 (4.05)	0.46 (0.01)	-1.02 (0.03)	6.95 (0.53)	34.14 (1.54)	0.19 (0.02)	0.09 (0.01)	47.58 (1.27)	22.2 (2.15)	0.71 (1.9)	0.3 (2.51)
		**RP - WS (*n* =8)**	15.44 (1.64)	0.09 (0.01)	165.6 (5.07)	0.33 (0.02)	-1.77 (0.12)	5.99 (1.18)	32.84 (2.22)	0.27 (0.04)	0.51 (0.11)	37.59 (1.96)	19.01 (2.55)	-2.21 (6.06)	-11.47 (12.13)
		**NRP - WW (*n* =7)**	28.28 (1.98)	0.22 (0.02)	131.93 (4.79)	0.43 (0.02)	-0.91 (0.05)	9.19 (1.19)	33.38 (2.33)	0.15 (0.02)	0.11 (0.02)	42.9 (2.67)	20.92 (2.58)	2.44 (2.9)	-5.17 (4.16)
		**NRP - WS (*n* =7)**	12.07 (1.32)	0.06 (0.01)	175.62 (6.49)	0.31 (0.02)	-1.97 (0.15)	4.27 (0.77)	30.64 (3.04)	0.38 (0.06)	0.63 (0.13)	34.41 (2.45)	19.4 (2.1)	3.27 (5.26)	-6.58 (13.96)
**SC103-14E**	**TIP 4.3 & 4.4**	**RP - WW (*n* =7)**	29.5 (1.44)	0.21 (0.01)	142.78 (4)	0.47 (0.01)	-0.96 (0.05)	7.87 (0.78)	34.6 (2.24)	0.17 (0.03)	0.09 (0.01)	47.68 (2.35)	23.29 (2.72)	2.92 (2.18)	5.2 (2.89)
		**RP - WS (*n* =3)**	11.36 (2.11)	0.07 (0.02)	176.33 (8.91)	0.34 (0.03)	-1.76 (0.17)	5.02 (1.03)	34.55 (4.42)	0.3 (0.08)	0.2 (0.08)	41.18 (3.86)	15.67 (0.93)	1.21 (5.03)	4.55 (7.5)
		**NRP - WW (*n* =7)**	30.63 (1.73)	0.23 (0.02)	137.96 (3.85)	0.42 (0.02)	-0.98 (0.05)	8.5 (0.82)	31.77 (2.1)	0.15 (0.02)	0.09 (0.01)	41.34 (1.96)	18.74 (2.51)	1.34 (2.78)	1.96 (2.67)
		**NRP - WS (*n* =3)**	10.99 (1.57)	0.06 (0.01)	186.86 (6.03)	0.31 (0.03)	-1.94 (0.19)	8.19 (2.1)	40.07 (3.3)	0.24 (0.07)	0.18 (0.07)	36.69 (3.19)	14.35 (2.05)	5.17 (4.83)	11.12 (3.71)
**IS9710**	**TIP 2.1**	**RP - WW (*n* =20)**	28.04 (1.16)	0.21 (0.01)	137.35 (2.88)	0.43 (0.01)	-0.98 (0.03)	6.95 (0.46)	33.04 (1.47)	0.21 (0.02)	0.1 (0.01)	42.09 (1.27)	21.18 (1.6)	4.26 (1.88)	-1.57 (2.13)
		**RP - WS (*n* =14)**	15.33 (1.23)	0.1 (0.01)	176.33 (5.36)	0.31 (0.02)	-1.8 (0.1)	5.31 (0.6)	32.01 (1.8)	0.33 (0.05)	0.28 (0.09)	32.79 (1.75)	17.62 (1.59)	-4.15 (3.28)	9.91 (3.61)
		**NRP - WW (*n* =5)**	29.95 (1.87)	0.24 (0.02)	130.33 (5.07)	0.4 (0.02)	-0.98 (0.06)	7.46 (0.68)	29.39 (2.75)	0.16 (0.02)	0.06 (0.03)	42.57 (2.29)	18.92 (3.71)	0.06 (2.8)	-4.4 (4.3)
		**NRP - WS (*n* =2)**	16.03 (2.7)	0.1 (0.02)	169.83 (7.54)	0.36 (0.05)	-1.57 (0.18)	4.49 (0.95)	36.38 (4.51)	0.29 (0.06)	0.21 (0.07)	33.9 (3.75)	14.69 (4.11)	-7.75 (6.28)	7.01 (5.33)
**Ai4**	**PIP 2.10**	**RP - WW (*n* =6)**	26.18 (1.76)	0.22 (0.02)	128.53 (5.31)	0.44 (0.02)	-1.03 (0.03)	8.57 (1.3)	33.55 (1.71)	0.17 (0.02)	0.08 (0.02)	41.57 (1.83)	18.46 (2.9)	2.6 (2.36)	-8.73 (4.58)
		**RP - WS (*n* =5)**	15.31 (1.46)	0.1 (0.01)	164.67 (8.53)	0.27 (0.02)	-1.77 (0.18)	6.58 (1.4)	28.87 (2.61)	0.41 (0.15)	0.54 (0.23)	28.56 (2.71)	13.8 (1.47)	-8.67 (4.59)	-9.89 (12.26)
		**NRP - WW (*n* =4)**	32.11 (1.48)	0.25 (0.01)	128.79 (3.37)	0.39 (0.03)	-1.07 (0.06)	11.21 (1.73)	29.32 (2.88)	0.12 (0.03)	0.1 (0.02)	43.47 (2.94)	19.21 (4.49)	-3.02 (1.74)	-2.85 (3.03)
		**NRP - WS (*n* =4)**	13.71 (2.68)	0.09 (0.02)	174.84 (7.84)	0.29 (0.04)	-1.52 (0.13)	4.86 (1.01)	29.28 (3.93)	0.32 (0.07)	-0.04 (0.07)	30.43 (4.13)	12.68 (1.92)	1.42 (9.61)	-8.45 (15.54)
**IS9710**	**PIP 1.6**	**RP - WW (*n* =14)**	28.72 (1.3)	0.22 (0.01)	134.81 (2.91)	0.41 (0.01)	-0.99 (0.03)	7.04 (0.48)	32.88 (1.68)	0.19 (0.02)	0.1 (0.02)	42.4 (1.41)	20.59 (1.96)	3.17 (2.08)	-3.02 (2.39)
		**RP - WS (*n* =8)**	16.46 (1.75)	0.1 (0.01)	177.16 (6.19)	0.32 (0.02)	-1.74 (0.14)	5.52 (0.6)	34.49 (2.04)	0.28 (0.07)	0.31 (0.11)	34.04 (2.26)	19.53 (2.18)	-6.13 (4.22)	12.72 (4.88)
		**NRP - WW (*n* =11)**	28.08 (1.37)	0.22 (0.01)	137.26 (4.09)	0.45 (0.02)	-0.98 (0.03)	7.09 (0.59)	31.43 (1.84)	0.21 (0.03)	0.09 (0.02)	41.92 (1.61)	20.79 (2.03)	3.59 (2.22)	-0.99 (2.81)
		**NRP - WS (*n* =8)**	14.3 (1.43)	0.09 (0.01)	173.51 (7.28)	0.32 (0.02)	-1.79 (0.12)	4.89 (0.87)	30.57 (2.62)	0.36 (0.05)	0.24 (0.1)	31.82 (2.29)	14.92 (1.98)	-3.15 (4.23)	6.09 (4.17)
**IS9710**	**PIP 1.1**	**RP - WW (*n* =21)**	29.48 (0.98)	0.23 (0.01)	134.22 (2.63)	0.43 (0.01)	-0.99 (0.02)	7.12 (0.41)	31.17 (1.31)	0.19 (0.02)	0.09 (0.01)	42.66 (1.14)	18.68 (1.51)	1.49 (1.47)	-1.93 (2.01)
		**RP - WS (*n* =13)**	15.53 (1.21)	0.1 (0.01)	172.19 (4.94)	0.32 (0.02)	-1.73 (0.09)	5.13 (0.59)	31.2 (1.92)	0.31 (0.04)	0.22 (0.07)	33.63 (1.7)	15.54 (1.45)	-5.61 (3.19)	7.23 (2.92)
		**NRP - WW (*n* =4)**	22.71 (2.78)	0.17 (0.02)	144.81 (6.15)	0.41 (0.03)	-0.93 (0.08)	6.68 (1.07)	37.88 (3.49)	0.26 (0.07)	0.12 (0.04)	39.71 (3.05)	31.12 (3.01)	13.78 (5.15)	-3.63 (4.79)
		**NRP - WS (*n* =3)**	15.01 (2.61)	0.09 (0.02)	190.88 (11.39)	0.29 (0.03)	-1.93 (0.22)	5.55 (1.04)	38.19 (1.97)	0.4 (0.17)	0.53 (0.23)	29.9 (3.78)	24.87 (3.68)	-0.15 (6.72)	20.46 (10.5)

Abbrevations:- A_n: Carbon assimilation rate; g_s: Stomatal conductance; *iWUE* : Instantaneous water use efficiency; $iWUE_{pc}$: *iWUE* attributed to variation in A_n; $iWUEg_s$: *iWUE* attributed to variation in g_s; *ΦPSII* : Operating quantum yield of photosystem II; Ψ_{leaf}: Midday leaf water potenial; K_{leaf}: Leaf hydraulic conductivity; *RWC* : Relative water content; *LMA* : Leaf mass per area; *LW* : Leaf width; R_{leaf}: hydraulic resistance of plant leaf; R_{rest}: hydraulic resistance of rest of the plant; SPAD: Relative chlorophyll content using SPAD; *Panicle* : Panicle size per plant; *Veg Biom:* Total biomass of vegetative parts per plant; *Tot Biom* : Total biomass of all parts per plant; $\Delta iWUE_{pc}$: *iWUE* attributed to variation in A_n; $\Delta iWUE_{gs}$: *iWUE* attributed to variation in g_s.

Table S4. ANOVA comparison between the three glasshouse chambers for some parameters.

Trait	Chamber Effect		
	df	*F-Stat*	*P-value*
g_s (mol m^{-2} s^{-1})	2	0.8664	0.4212
**Flowering date (week)**	2	1.0401	0.3543
**Plant Height (cm)**	2	0.4495	0.6383
Vegetative Biomass **(g plant^{-1})**	2	2.1174	0.1215
$\Psi_{pre\text{-}dawn}$ (-MPa)	2	1.0109	0.3647

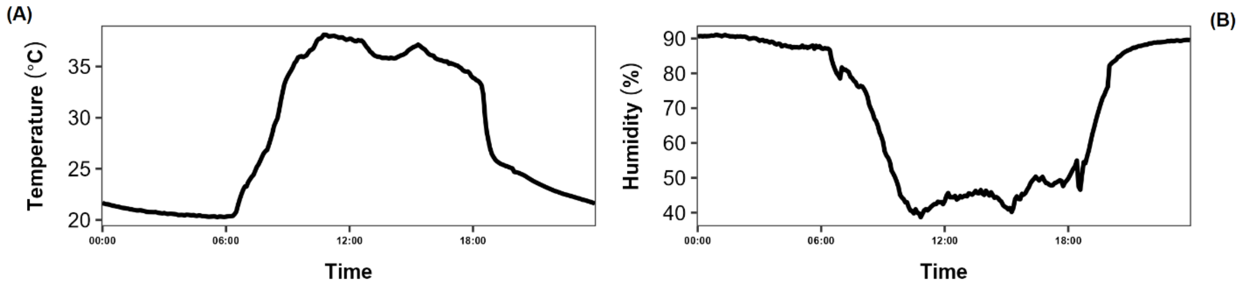

Fig. S1 Average diurnal conditions of the three glasshouses used measured using a tinytag logger.

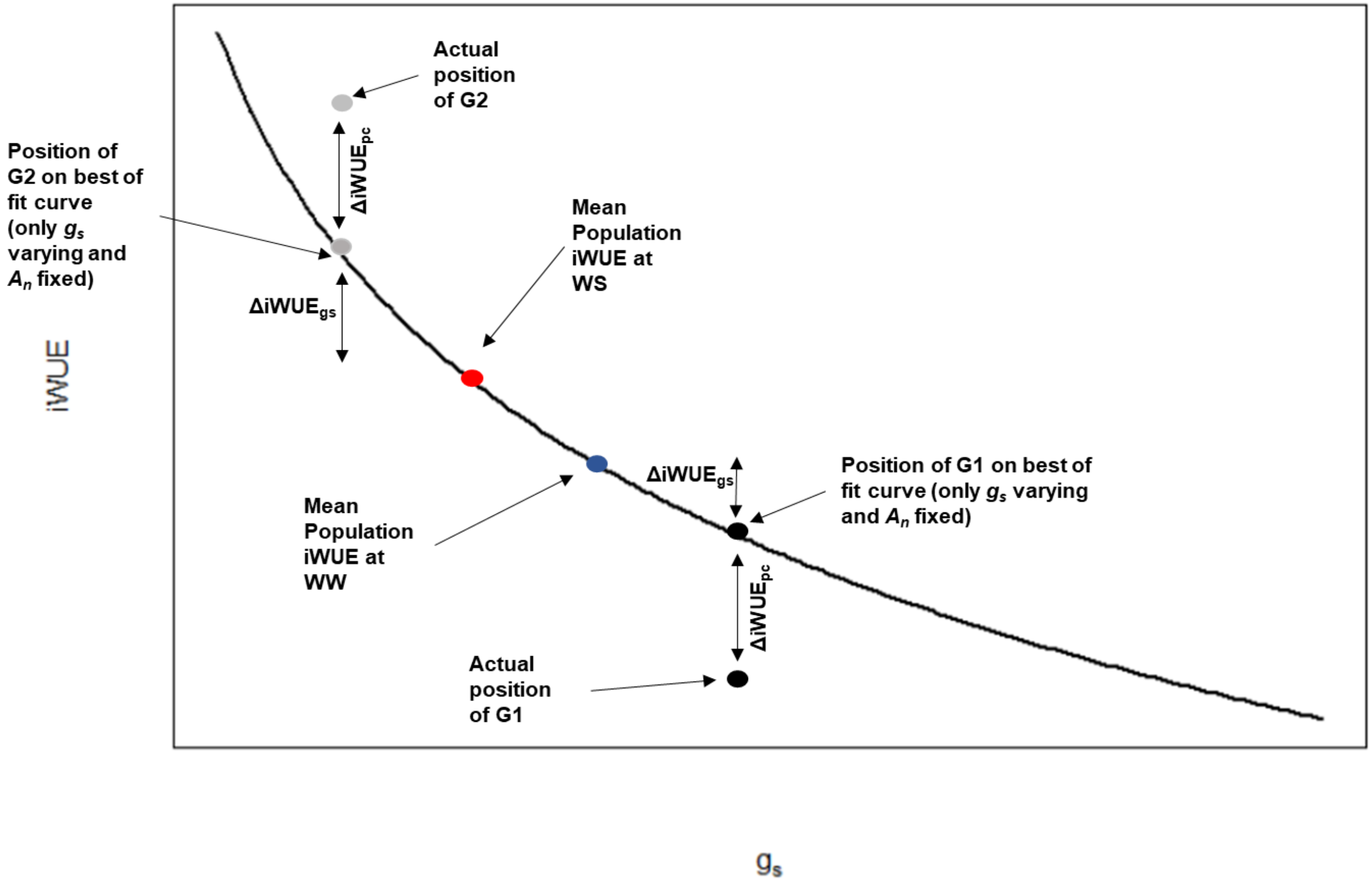

The curve represents the idealized intrinsic water use efficiency (*iWUE*) vs stomatal conductance (g_s) relationship, with this relationship being we can quantify effect of g_s on *iWUE* irrespective of A_n. Hence, the position of the genotype on the idealized curve from the mean *iWUE* represents the effect of g_s on *iWUE, ΔiWUE$_{gs}$*. The remaining difference between the position of the genotype on the idealized curve and its actual position within *iWUE*- g_s distribution is the effect of A_n, *ΔiWUE$_{pc}$*.

The example genotypes show extremes:
G1 – is a genotype with high g_s but A_n does not compensate for that in terms of *iWUE*, reducing its *iWUE* below the curve. This genotype might be water-stress sensitive.

G2 – is a genotype with low g_s but A_n is proportionally higher for that g_s value leading to actual *iWUE* that is higher than the one on the idealized curve. This genotype can maybe maintain photosynthesis despite low g_s under water-stress.

Fig. S2 Figure showing how the components of intrinsic water use efficiency (*iWUE*) were calculated and what do they mean in terms of net carbon assimilation (A_n) vs stomatal conductance (g_s) interactions.

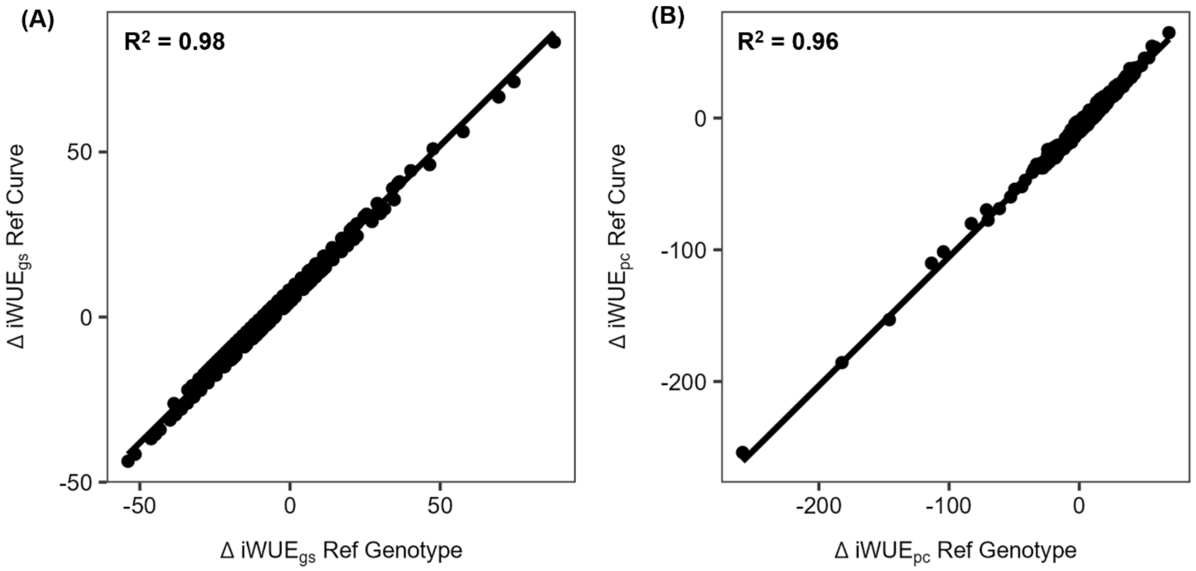

Fig. S3 The relationship between the components of iWUE as calculated via a reference curve compromising the entire population of genotypes (Our methods or as (Li *et al.*, 2017)) and calculated via a reference genotype as per (Gilbert *et al.*, 2011).

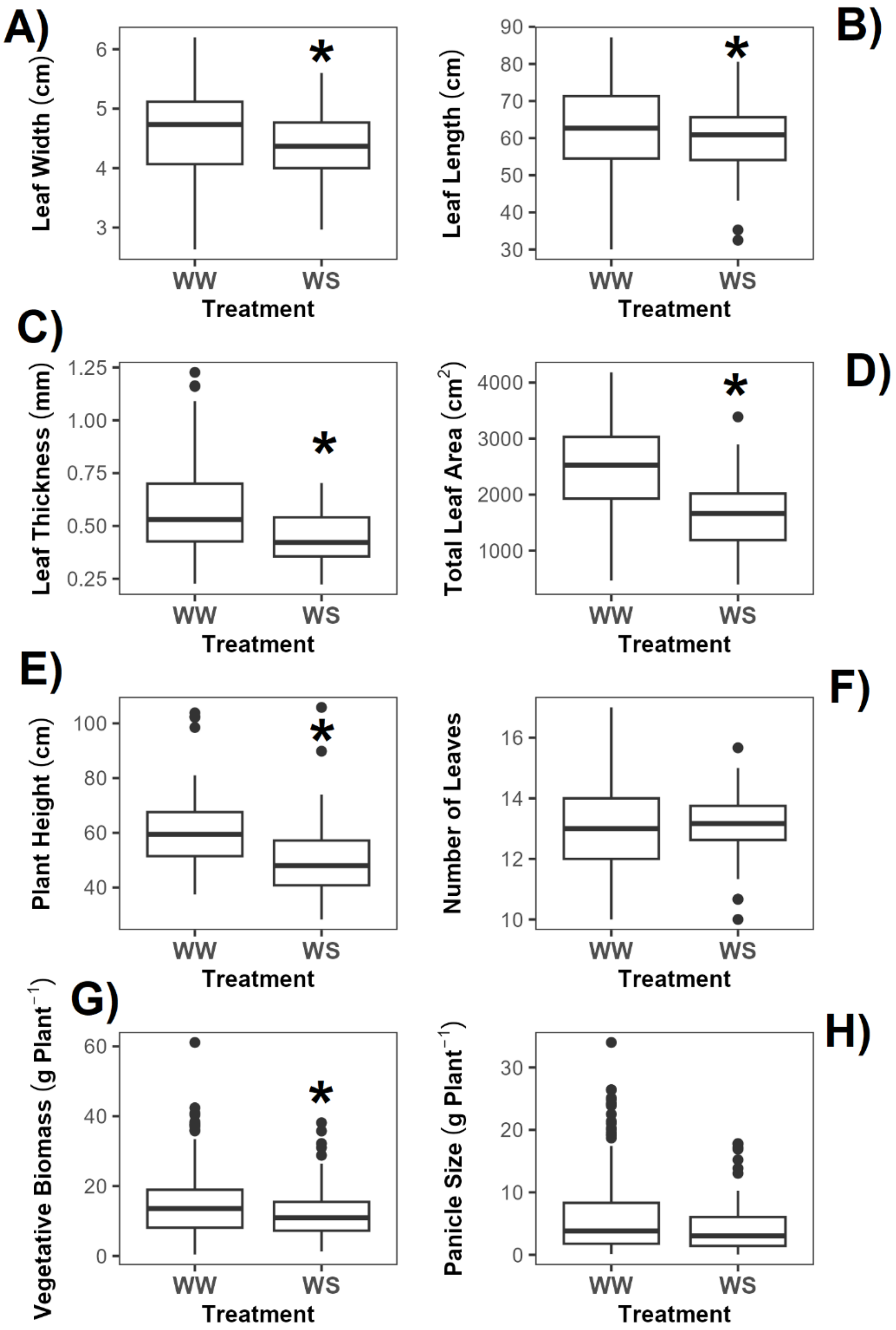

Fig.S4 The distribution of leaf morphological and harvest characteristics within the two watering treatments. The distribution is summarized by boxplots for each treatment. The values in each distribution compromise the mean of every genotype (*n*=3) per treatment, yielding *n*=89 for Well-Watered treatment and *n*=60 for Water Stress treatment. Each box encompasses the 25th and 75th percentiles, with whiskers extending to show the extremes. Statistically significant difference is represented at the top of each boxplot with * indication to highlight a *P*-value of 0.05 or lower. (A) Leaf Width; (B) Leaf Length; (C) Leaf Thickness; (D) Total Leaf Area; (E) Plant Height; (F) Number of Leaves; (G) Vegetative Biomass; (H) Panicle Size.

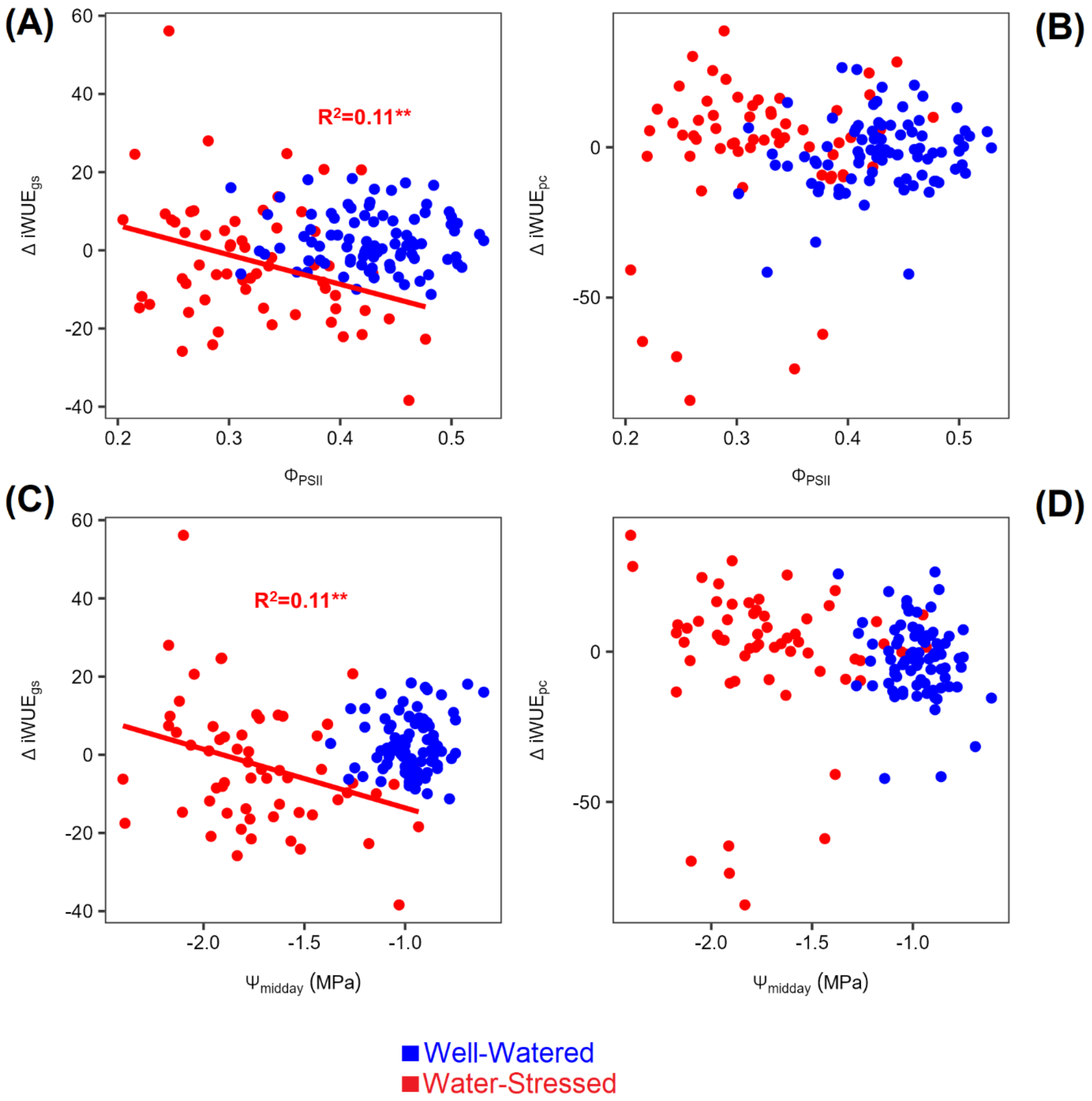

Fig.S5 Relationship between components of intrinsic water use efficiency and leaf water potential and photosynthetic efficiency. Each point in scatter plots represent genotype average (*n*=3). Standard error was removed to provide clearer presentation and are presented in Table S1. The regression line represents the line of best fit for the data (or part of the data), with the corresponding R^2 value from a Pearson product-moment correlation analysis (Table S1). Degrees of statistical significance are represented as: *P*<0.001 (***), *P*<0.05 (**). **(A)** variation in *iWUE* due to stomatal conductance ($\Delta iWUE_{gs}$) vs Operating efficiency of Photosystem II (Φ_{PSII}); **(B)** Variation in *iWUE* due to photosynthetic capacity ($\Delta iWUE_{pc}$) vs Φ_{PSII}; **(C)** $\Delta iWUE_{gs}$ vs Midday leaf water potential (Ψ_{midday}); **(D)** $\Delta iWUE_{pc}$ vs Ψ_{midday}.

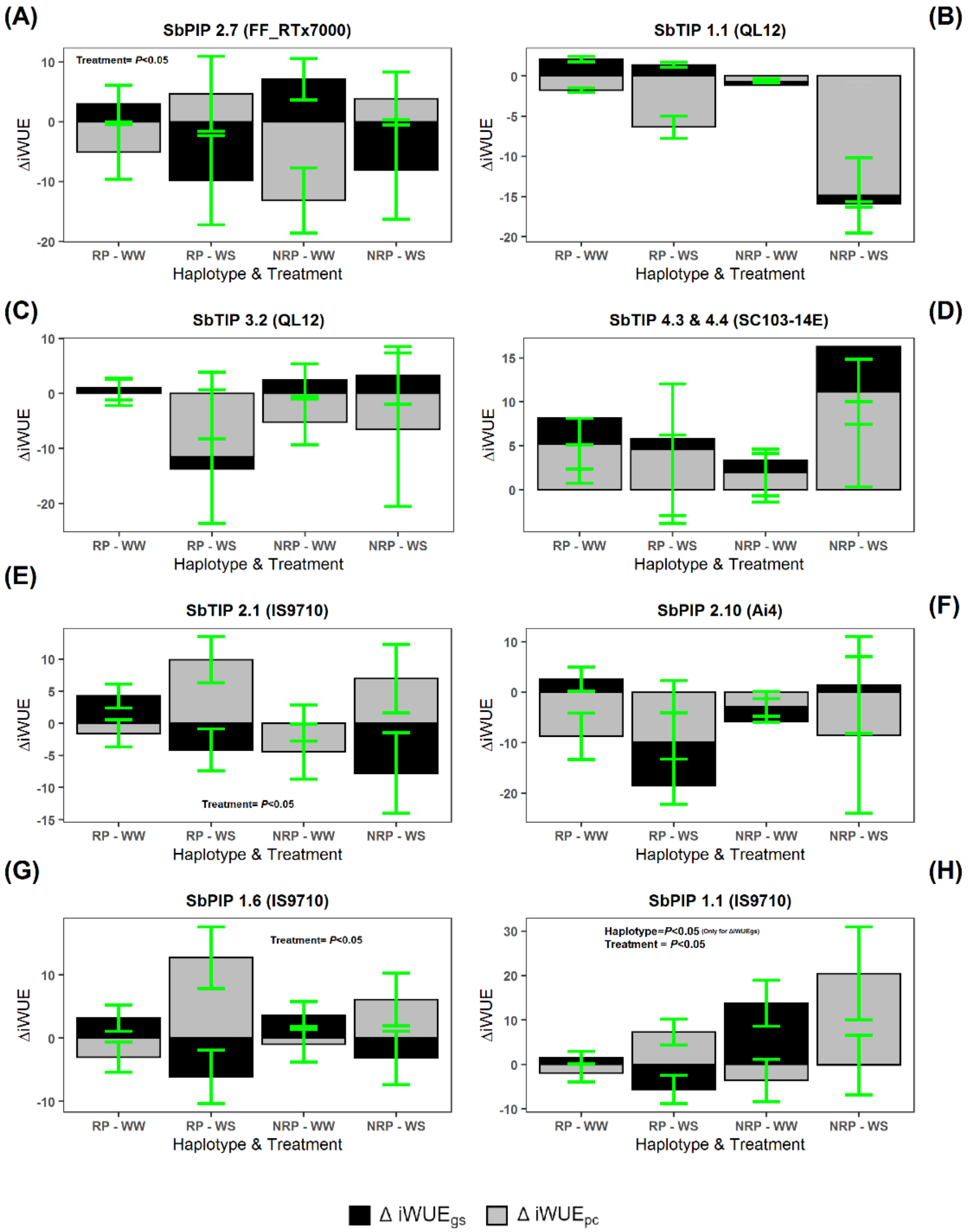

Fig.S6 Bar charts showing the effects of water stress on the contribution of variation in the two components of intrinsic water use efficiency (stomatal, $\Delta iWUE_{gs}$, and photosynthetic, $\Delta iWUEpc$) on the sorghum lines grouped by identified aquaporin. The full analysis for all AQPs and traits is shown in Table S3. Each bar represents the mean of all individual replicates belonging to the genotypes of that population (*n*=6-63; see Table S3 for the number of genotypes for each Haplotype * Treatment combination). Each population (RP & NRP) refers to a set of genotypes that have either inherited the AQP haplotype block from the elite parent (RP) or from the exotic parent (NRP). **(A)** - **(G)** plots represent the different aquaporin groups. Error bars are standard error.

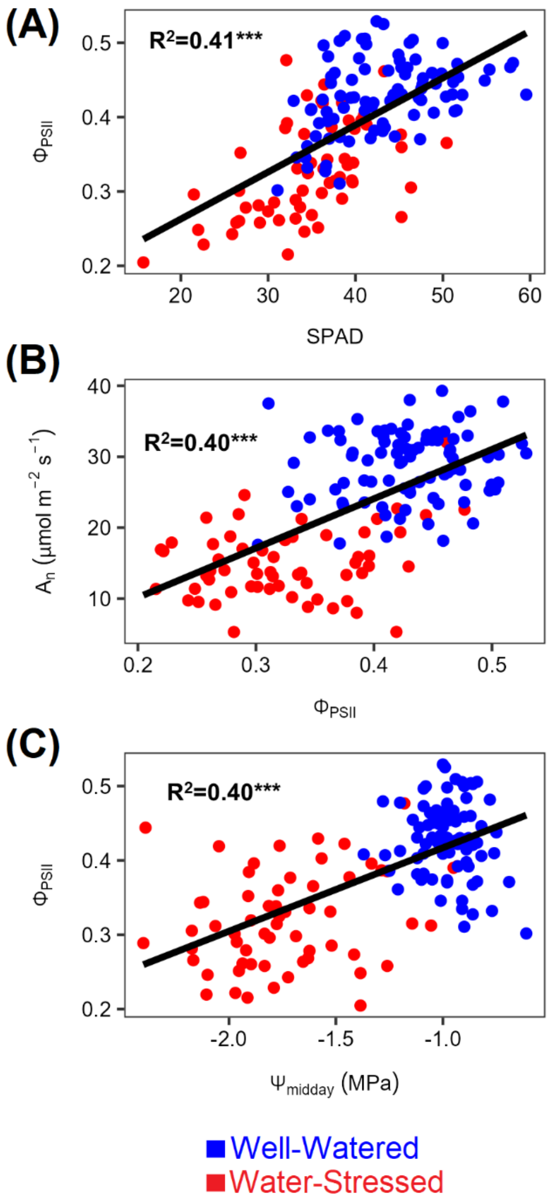

Fig.S7 Relationship between photosynthesis, chlorophyll content and leaf water potential. Each point in scatter plots represent genotype average (n=3). Standard error was removed to provide clearer presentation and are presented in Table S1. The regression line represents the line of best fit for the data, with the corresponding R^2 value from a Pearson product-moment correlation analysis. Degrees of statistical significance are represented as: P<0.001 (***), P<0.05 (**). **(A)** Operating efficiency of Photosystem II (Φ_{PSII}) vs Chlorophyll content (*SPAD*); **(B)** Carbon assimilation rate (A_n) vs Φ_{PSII}; **(C)** Φ_{PSII} vs Midday leaf water potential (Ψ_{midday}).

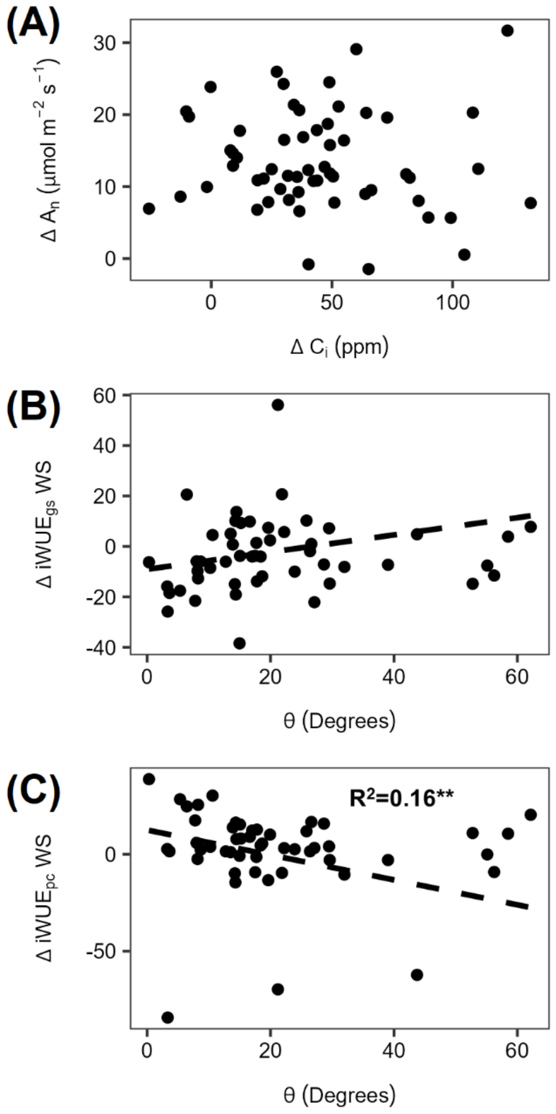

Fig.S8 Relationship between parameters describing the change in the carbon assimilation – intercellular CO_2 concentration relationship and components of intrinsic water use efficiency under water-stress. The regression line represents the line of best fit for the data (or part of the data), with the corresponding R^2 value from a Pearson product-moment correlation analysis (Table S2). Degrees of statistical significance are represented as: P<0.001 (***), P<0.05 (**). **(A)** Difference in carbon assimilation rate between well-watered and water-stressed (ΔA_n) vs Difference in operational intercellular CO_2 concentration between well-watered and water-stressed (ΔC_i); **(B)** variation in *iWUE* due to stomatal conductance under water-stress ($\Delta iWUE_{gs}$ WS) vs the inverse tan of ΔA_n / ΔC_i (θ); (C) variation in *iWUE* due to photosynthetic capacity under water-stress ($\Delta iWUE_{pc}$ WS) vs θ.

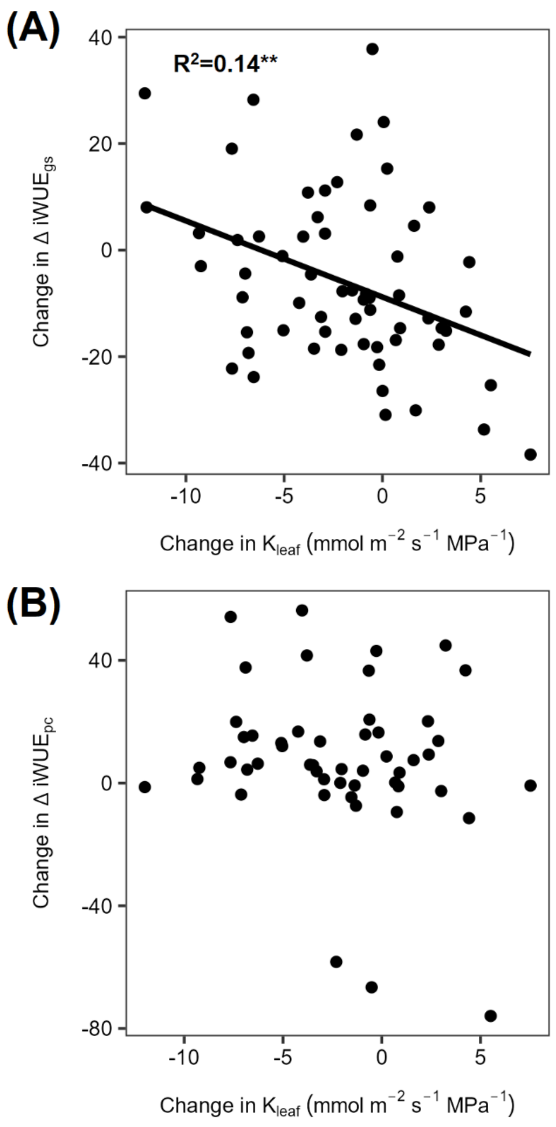

Fig.S9 (A) The difference in variation in *iWUE* due to stomatal conductance ($\Delta iWUE_{gs}$) between water-stressed and well watered (WS – WW) vs change in leaf hydraulic conductance (K_{leaf}) from water-stressed to well-watered (WS - WW); **(B)** The difference in variation in *iWUE* due to photosynthetic capacitty ($\Delta iWUE_{pc}$) between water-stressed and well watered (WS – WW) vs change in leaf hydraulic conductance (K_{leaf}) from water-stressed to well-watered (WS - WW). The regression line represents the line of best fit for the data (or part of the data), with the corresponding R^2 value from a Pearson product-moment correlation analysis (Table S1). Degrees of statistical significance are represented as: P<0.001 (***), P<0.05 (**).

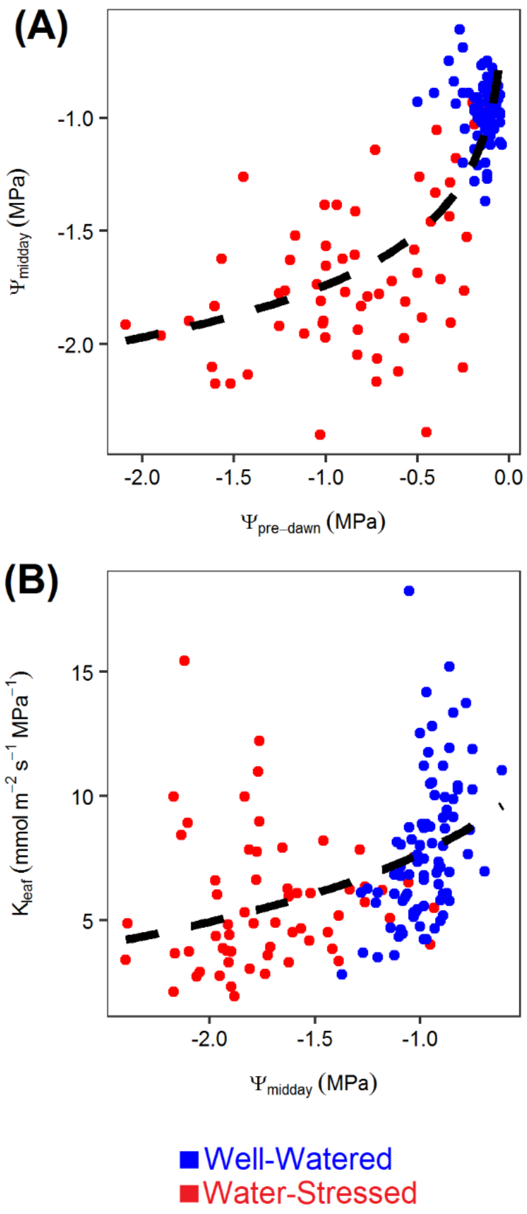

Fig.S10 Relationships between hydraulic conductivity & water potentials. Each point in scatter plots represent genotype average (*n*=3). Standard error was removed to provide clearer presentation and are presented in Table S1. The regression line represents the line of best fit for the data. **(A)** Midday leaf water potential (Ψ_{midday}) vs Pre-dawn leaf water potential ($\Psi_{pre\text{-}dawn}$); **(B)** Leaf hydraulic conductivity (K_{leaf}) vs Ψ_{midday}.

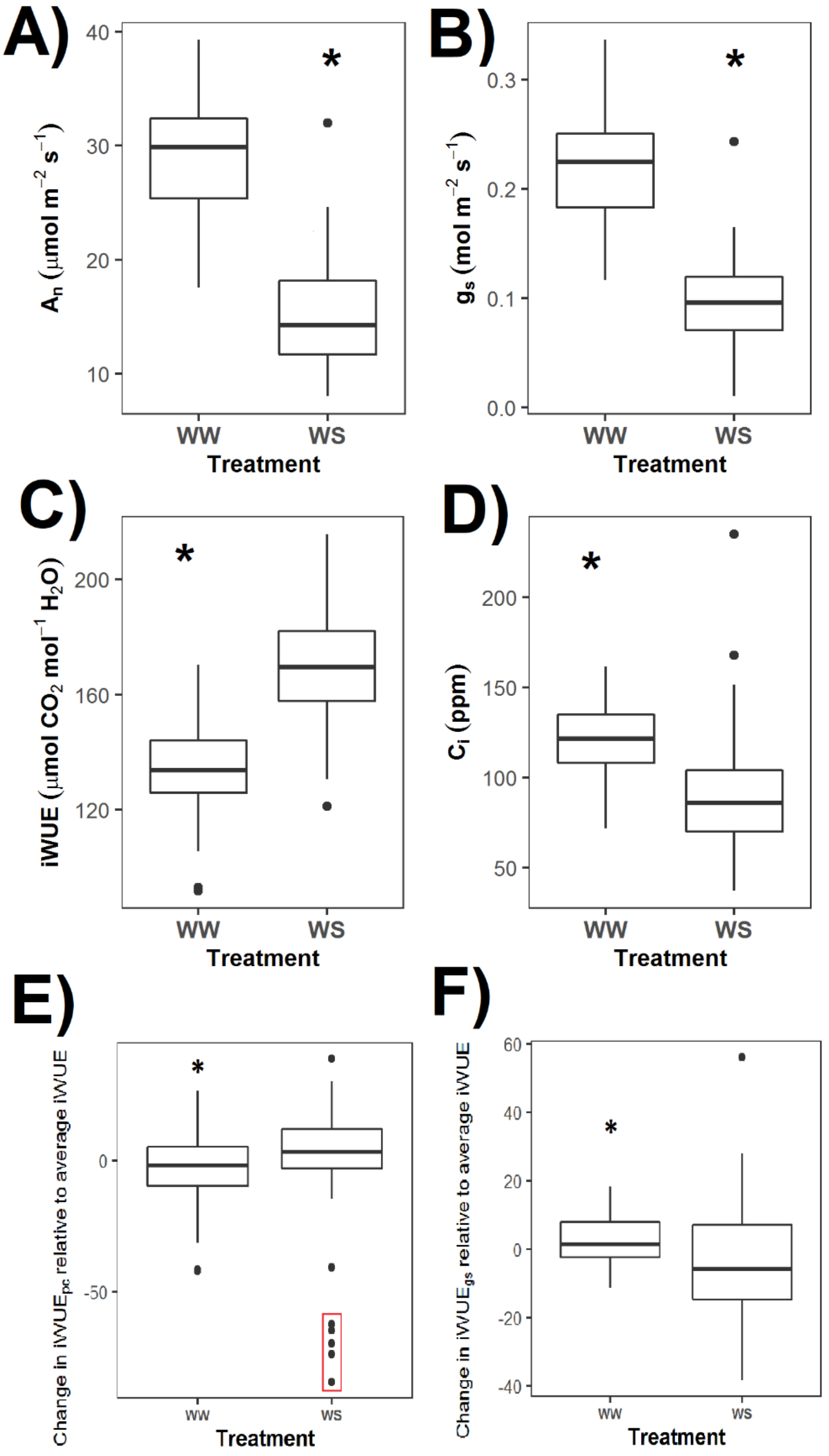

Fig.S11 The distribution of gas exchange values within the two watering treatments. The distribution is summarized by boxplots for each treatment. The values in each distribution compromise the mean of every genotype (n=3) per treatment, yielding *n*=89 for Well-Watered treatment and *n*=60 for Water Stress treatment. Each box encompasses the 25th and 75th percentiles, with whiskers extending to show the extremes. Statistically significant difference is represented at the top of each boxplot with ***** indication to highlight a *P*-value of 0.05 or lower. **(A)** Net carbon assimilation rate (A_n); **(B)** Stomatal conductance (g_s); **(C)** Intrinsic water use efficiency (*iWUE*); **(D)** Sub-stomatal carbon dioxide concentration (C_i); **(E)** Variation in *iWUE* due to photosynthetic capacity ($iWUE_{pc}$); **(F)** variation in *iWUE* due to stomatal conductance ($iWUE_{gs}$).

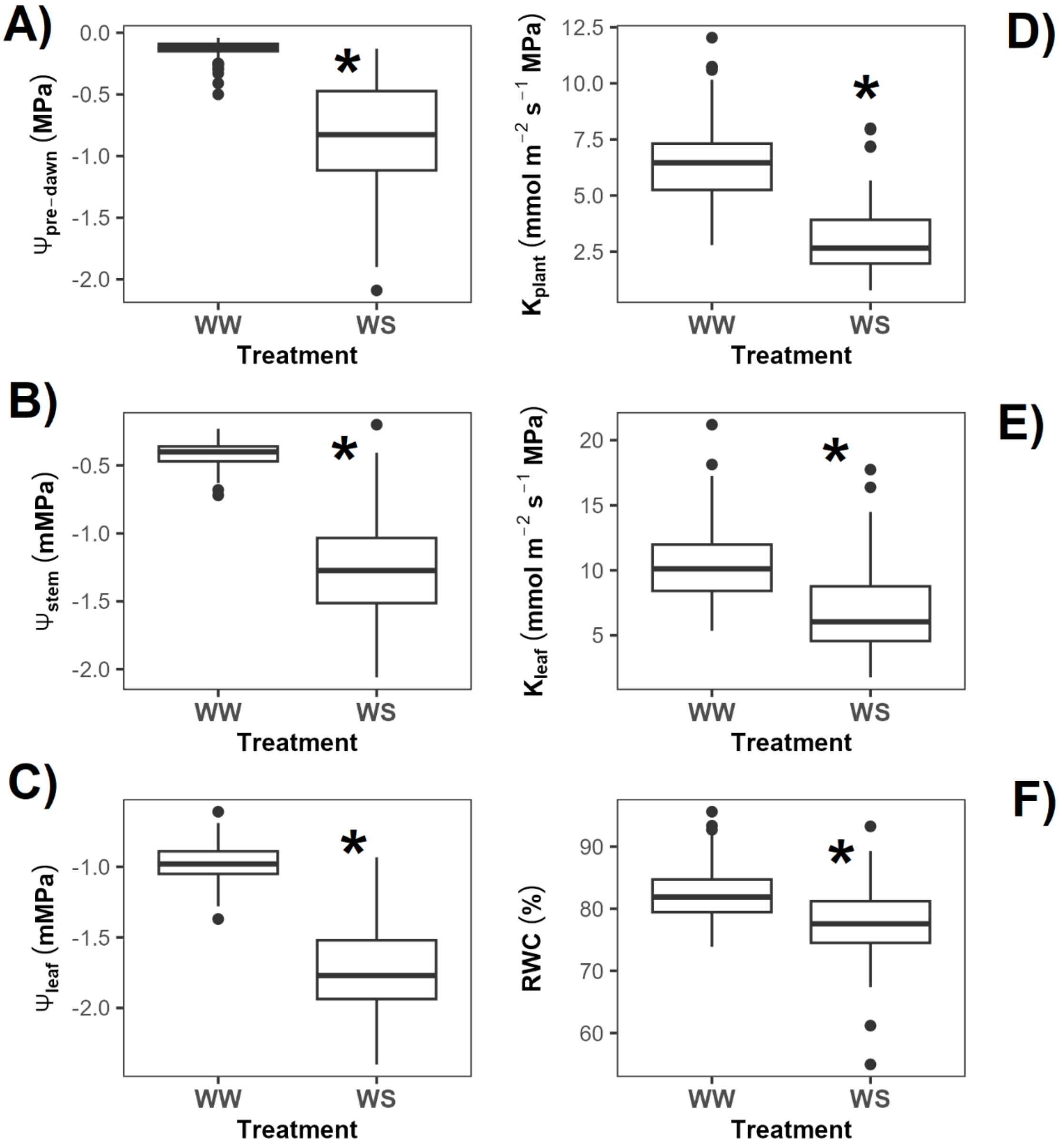

Fig.S12 The distribution of leaf and plant hydraulic characteristics within the two watering treatments. The distribution is summarized by boxplots for each treatment. The values in each distribution compromise the mean of every genotype (*n*=3) per treatment, yielding *n*=89 for Well-Watered treatment and *n*=60 for Water Stress treatment. Each box encompasses the 25th and 75th percentiles, with whiskers extending to show the extremes. Statistically significant difference is represented at the top of each boxplot with ***** indication to highlight a *P*-value of 0.05 or lower. (A) Pre-Dawn leaf water potential ($\Psi_{pre\text{-}dawn}$); (B) Midday stem water potential (Ψ_{stem}); (A) Midday leaf water potential (Ψ_{leaf}); (D) Plant hydraulic conductivity (K_{plant}); (E) Leaf-specific hydraulic conductivity (K_{leaf}). (F) Relative water content (*RWC*).

REFERENCES

Gilbert ME, Zwieniecki MA, Holbrook NM. 2011. Independent variation in photosynthetic capacity and stomatal conductance leads to differences in intrinsic water use efficiency in 11 Soybean genotypes before and during mild drought. Journal of Experimental Botany **62**, 2875–2887.

Li C, Jackson P, Lu X, Xu C, Cai Q, Basnayake J, Lakshmanan P, Ghannoum O, Fan Y. 2017. Genotypic variation in transpiration efficiency due to differences in photosynthetic capacity among sugarcane-related clones. Journal of Experimental Botany **68**, 2377–2385.
